# Genome-wide gene-air pollution interaction analysis of lung function in 300,000 individuals

**DOI:** 10.1016/j.envint.2021.107041

**Published:** 2022-01-15

**Authors:** Carl A. Melbourne, A. Mesut Erzurumluoglu, Nick Shrine, Jing Chen, Martin D. Tobin, Anna L. Hansell, Louise V. Wain

**Affiliations:** a Department of Health Sciences, University of Leicester, Leicester, UK; b MRC Epidemiology Unit, University of Cambridge, Cambridge, UK; c National Institute for Health Research, Leicester Respiratory Biomedical Research Centre, Glenfield Hospital, Leicester, UK; d Centre for Environmental Health and Sustainability, University of Leicester, Leicester, UK; e National Institute for Health Research Health Protection Research Unit in Environmental Exposures and Health at the University of Leicester, Leicester, UK

**Keywords:** Gene-air pollution interactions, Air pollution, Genome-wide, Lung function, Chronic Obstructive Pulmonary Disease, UK Biobank

## Abstract

**Background:**

Impaired lung function is predictive of mortality and is a key component of chronic obstructive pulmonary disease. Lung function has a strong genetic component but is also affected by environmental factors such as increased exposure to air pollution, but the effect of their interactions is not well understood.

**Objectives:**

To identify interactions between genetic variants and air pollution measures which affect COPD risk and lung function. Additionally, to determine whether previously identified lung function genetic association signals showed evidence of interaction with air pollution, considering both individual effects and combined effects using a genetic risk score (GRS).

**Methods:**

We conducted a genome-wide gene-air pollution interaction analysis of spirometry measures with three measures of air pollution at home address: particulate matter (PM_2.5_ & PM_10_) and nitrogen dioxide (NO_2_), in approximately 300,000 unrelated European individuals from UK Biobank. We explored air pollution interactions with previously identified lung function signals and determined their combined interaction effect using a GRS.

**Results:**

We identified seven new genome-wide interaction signals ($P\;<\;5\times 10^{-8}$), and a further ten suggestive interaction signals ($P\;<\;5\times 10^{-7}$). Additionally, we found statistical evidence of interaction for FEV_1_/FVC between PM_2.5_ and previously identified lung function signal, rs10841302, near *AEBP2*, suggesting increased susceptibility as copies of the G allele increased (but size of the impact was small - interaction beta: -0.363 percentage points, 95% CI: -0.523, -0.203 per 5 µg/m^3^). There was no observed interaction between air pollutants and the weighted GRS.

**Discussion:**

We carried out the largest genome-wide gene-air pollution interaction study of lung function and identified potential effects of clinically relevant size and significance. We observed up to 440 ml lower lung function for certain genotypes when exposed to mean levels of outdoor air pollution, which is approximately equivalent to nine years of average normal loss of lung function in adults.

## Highlights

• Gene-air pollution interaction effects on lung function are poorly understood.
• Seven new potential gene-air pollution interaction signals identified.
• Interaction effects identified are large enough to be clinically relevant.
• Observed interactions include one previously identified lung function signal.
• High-risk genetic subgroups potentially more susceptible to outdoor air pollution.

## Graphical abstract

**Figure:**
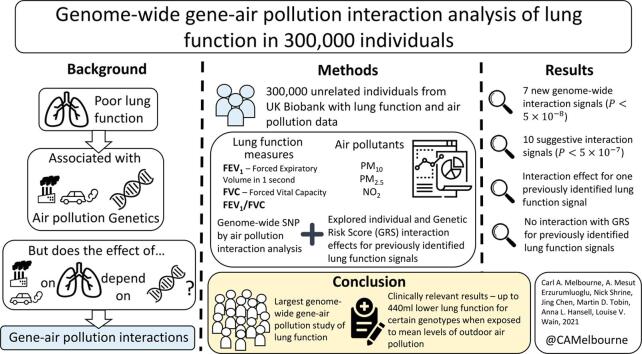

## 1 Introduction

Impaired lung function is predictive of mortality and is a key component in the diagnosis of chronic obstructive pulmonary disease (COPD). Smoking is the biggest risk factor for COPD, which is thought to have caused as many as 2.9 million deaths worldwide in 2016 (GBD 2016 Causes of Death Collaborators, 2017) although other sources of indoor air pollution are also associated with COPD risk (Rabe & Watz, 2017; Agustí & Hogg, 2019). Furthermore, increased exposure to air pollution is associated with lower lung function (Doiron, et al., 2019).

Lung function and COPD risk is also influenced by genetic factors and we and others have discovered over 300 genetic association signals for COPD risk and/or lung function measures (Sakornsakolpat, 2019, Shrine, 2019). Combining these signals into a single genetic risk score, we have previously shown that individuals in the highest decile of genetic risk have an almost 5-fold increased risk of COPD compared to those in the lowest decile (Shrine et al., 2019). However, collectively, these variants only explain up to around 13% of the heritability of lung function.

We hypothesised that there could be interactions between genetic variants and air pollution measures which affect COPD risk and lung function. Detection of such effects could enable identification of high-risk subgroups of the population and provide new biological insight into the mechanisms whereby air pollution affects respiratory health.

To test this hypothesis, we carried out the largest genome-wide gene-air pollution interaction study of lung function in ∼ 300,000 individuals from UK Biobank, using particulate matter (PM) and nitrogen dioxide (NO_2_) concentrations as measures of air pollution exposure.

## 2 Methods

### 2.1 Study participants

We used spirometry, anthropometric, questionnaire and genetic data for individuals in UK Biobank, collected at baseline (upon recruitment) between 2006 and 2010. UK Biobank is a large-scale research database, containing both genetic and health information for a national cohort of over 500,000 individuals aged between 40 and 69 years.

### 2.2 Selection of individuals with lung function data

We selected unrelated European individuals from UK Biobank as previously described (Shrine et al., 2019). In summary, we selected individuals that had complete lung function data and passed our previously outlined quality control filters (N = 348,936) for forced expiratory volume in 1 second (FEV_1_), forced vital capacity (FVC) and the ratio (FEV_1_/FVC). From this we then selected a subsample of unrelated individuals (N = 303,320) of genetically determined European ancestry (KING kinship coefficient < 0.0884 corresponding to below 2nd degree kinship (Manichaikul et al., 2010)). All individuals had complete data for sex, age, height and ever smoking status (ever vs never).

### 2.3 Air pollution data

Air pollution concentrations at place of residence of UK Biobank participants at recruitment (at time of pulmonary function testing) were estimated using European Study of Cohorts and Air Pollution Effects (ESCAPE) land use regression models (Eeftens et al., 2012, Beelen et al., 2013). In these analyses, we explored associations with fine particles with average diameter < 2.5 µm (PM_2.5_), particulate matter with average aerodynamic diameter < 10 µm (PM_10_) and annual average concentrations of nitrogen dioxide (NO_2_).

ESCAPE model predictions were compared to the UK’s Automative Urban and Rural Network (AURN) data (Gulliver & Hoogh, 2015) to evaluate air pollution model estimates. NO_2_ concentrations were predicted reasonably well throughout the country (R^2^ = 0.67). PM_10_ concentrations were moderately well estimated for central and southern UK areas (R^2^ = 0.53) but less so for nothern England or Scotland (R^2^ < 0.5) as models were not robust > 400 km from Greater London. The PM analyses therefore did not include participants from northern England and Scotland [see (Doiron et al., 2017) for more details on air pollution concentration modelling].

### 2.4 Genome-wide interaction analysis

FEV_1_, FVC and FEV_1_/FVC were adjusted for sex, age, age^2^, height and ever smoking. Residuals were then inverse normal transformed.

Individuals were genotyped using the Affymetrix Axiom UK BiLEVE and Affymetrix Axiom UK Biobank arrays (Bycroft et al., 2018) with imputation undertaken using the Haplotype Reference Consortium (HRC) (McCarthy et al., 2016) and combined UK10K + 1000 genomes (Huang et al., 2015) reference panels. Multiallelic variants were removed and variants imputed with low confidence were excluded (imputation quality r^2^ < 0.5 for all SNPs and r^2^ < 0.8 for rare SNPs with minor allele frequency (MAF) < 1%). Variants with MAF < 0.5% were removed.

Each transformed lung function trait was used as the outcome in a multiple regression model which included the first 15 principal component terms for ancestry, genotyping array, SNP term (using an additive genetic model), air pollution variable and an interaction term for the interaction between SNP and air pollution:

$${\mathrm{Phenotype}}_{i}=\beta_{0}+\beta_{1}G_{i}+\beta_{2}A_{i}+\beta_{3}G_{i}A_{i}+{\mathrm{PC}}_{1i}{\cdots {\mathrm{PC}}_{15i}+{\mathrm{Array}}_{i}+\varepsilon }_{i}$$

where $G_{i}$ is the genotype for individual i, $A_{i}$ is the air pollution value, ${\mathrm{PC}}_{1i}\cdots {\mathrm{PC}}_{15i}$ represent principal component values and ${\mathrm{Array}}_{i}$ is the genotype array value (coded 0 and 1 for UK Biobank array and UK BiLEVE array respectively). The p-value returned for the $\beta_{3}$ estimate corresponds to the interaction effect between SNP and air pollution value ($G_{i}A_{i}$). Multiple regression was performed using PLINK2 (Chang et al., 2015).

Air pollution measures PM_2.5_ and PM_10_ were transformed into standard z-scores due to observed collinearity issues as a result of strong correlation between the air pollution variable ($A_{i}$) and interaction ($G_{i}A_{i}$) in the regression model (observed due to small variances for air pollution measures PM_2.5_ and PM_10_, **Supplementary Fig. 1**). Air pollution measure NO_2_ was analysed untransformed.

### 2.5 Signal selection and signal refinement

To define association signals and their sentinel variants, all variants were ranked by p-value and the SNP with the lowest p-value was selected as the first signal sentinel. All SNPs +/−1 megabase (Mb) either side of this first sentinel were then excluded and the process repeated for the next most significant SNP until all 2 Mb regions containing a sentinel SNP with $P\;<\;5\times 10^{-8}$ had been identified (genome-wide signals). The process was repeated to define a set of signals with sentinel SNPs at threshold of $P\;<\;5\times 10^{-7}$ (suggestive signals). Conditional analysis was used to identify additional independent genome-wide and suggestive signals by including the sentinel interaction term in the model, re-analysing all SNPs within each 2 Mb region and determining whether any SNPs remained below the pre-specified threshold. Region plots for each signal were created using LocusZoom (Pruim et al., 2010).

To aid the interpretation of interaction effects for genome-wide significant interaction signals, we presented the association between lung function trait and air pollution variable stratified by genotype group. To do this, dosages were converted to direct genotype calls by rounding to the nearest genotype group.

Using a Bayesian method (Wakefield, 2007) we refined each signal to a credible set of SNPs (the set of SNPs 95% likely to contain the causal SNP, under the assumption that the causal SNP was analysed).

### 2.6 Identification of putative causal genes

Credible set SNPs including the sentinel SNP were annotated using Annovar (Wang, et al., 2010) to identify coding variants with a putative functional effect (for example, missense). To identify whether any of the signals were independently associated with gene expression, we searched the GTEx (GTEx Consortium, 2013) and blood eQTLgen (Westra et al., 2013) eQTL catalogues. To identify a potential shared causal variant between the SNP-air pollution interaction signals and the eQTL gene expression signals, colocalisation was undertaken using COLOC (Giambartolomei et al., 2014) where full summary data was available in GTEx and eQTLgen databases (Võsa et al., 2021). An observed probability > 0.8 for a shared causal variant was used as the threshold to conclude colocalisation of SNP-air pollution and gene expression signals. We queried the sentinel SNPs in Open Target Genetics (Ghoussaini et al., 2021) for eQTL associations (which in addition to GTEx includes a further 14 consortia with eQTL expression association results) and to identify associations with protein expression (pQTL) and overlap with regions known to interact with gene promoters (promotor capture HiC).

### 2.7 Association with other phenotypes

The SNP with the highest posterior probability for causality in each credible set was queried in PhenoScanner (Staley et al., 2016) and Open Targets Genetics (Ghoussaini et al., 2021) resources to identify shared associations with other phenotypes at a threshold of $P\;<\;1\times 10^{-3}$.

### 2.8 Tissue-specificity of interaction signals

To identify whether there was enrichment of SNP-air pollution interaction signals within regulatory regions of the genome (for example, DNase I Hypersensitive Sites (DHS)) in specific cell or tissue types we used GARFIELD (Iotchkova et al., 2019). The software determines whether signals are enriched for DHS across 55 tissues (with an adjusted significant enrichment threshold for 540 effective annotations of  $P\;<\;9.26\times 10^{-5}$). We investigated the functional impact of SNPs (potential chromatin effects) which were highly probable to be the drivers of each signal (i.e. SNPs with posterior probability > 0.9 in credible sets) using DeepSEA (Zhou & Troyanskaya, 2015). To define a significant functional impact we used an E-value < 0.05 (the proportion of 1000 Genomes SNPs predicted to have a higher magnitude for chromatin effect compared to the chosen SNP being investigated) and an absolute probability difference > 0.1 between alternative and reference allele (the threshold defined for ‘high confidence’).

### 2.9 Sensitivity analyses

#### 2.9.1 Effect of Socio-Economic status

Socio-economic status (SES) of an individual is a plausible moderator of lung function, with observed modification of air pollution effects (Doiron et al., 2019), however adjusting for SES in our analyses would have led to a reduction of approximately 13% in the discovery sample size due to missing data. We accounted for any effects of SES on genome-wide interaction signals in two ways. Firstly, we undertook a sensitivity analysis for the top signals adjusting for educational status and income status using a complete-case analysis (after inverse normalisation of lung function traits). Secondly, we present interaction effects for genome-wide signals across categorised groups for income and educational status to visualise any difference in effect (akin to a three-way interaction between SNP, air pollutant and education/income). Income status was categorised using the definition in UK Biobank of “less than £18,000”, “£18,000 to £30,999”, “£31,000 to £51,999”, “£52,000 to 100,000” and “> 100,000”. Educational status was dichotomised as “lower vocational qualification or less” vs “higher vocational qualification or more”, grouping A-level (2), O-level (3), CSEs (4), and “None of the above” (−7) under “low education”, and College/University (1), NVQ (5) and Other professional qualifications (6) under “high education”. Individuals who selected “Do not know” (−1), “Prefer not to answer” (−3) or have missing data were excluded from subsequent analyses.

#### 2.9.2 Exposure misspecification

Misspecification of a continuous exposure in statistical models, such as incorrectly modelling non-linear effects as linear, has been shown to inflate type I error rates when studying gene-environment interactions leading to identification of false-positives (Tchetgen Tchetgen and Kraft, 2011, Sun et al., 2018). To determine whether this affected our conclusions (estimates and statistical significance), we re-calculated interaction effects for genome-wide gene-air pollution interaction signals using the same statistical model as before with inclusion of non-linear terms (quadratic and cubic to model the air pollution effect).

### 2.10 Previously reported lung function and COPD association signals

We performed a look-up in the genome-wide gene-air pollution interaction analyses (for all three air pollution measures and all three lung function measures), for the 304 signals previously reported for association with lung function and COPD (279 lung function signals from Shrine et al. 2019 (Shrine et al., 2019) and 25 signals from Sakornsakolpat et al. 2019 (Sakornsakolpat et al., 2019)). As these independent signals have *a priori* evidence for association with lung function or COPD, we applied a Bonferroni corrected threshold for 304 tests to define a significant air pollution interaction effect ($P\;<\;1.6\times 10^{-4}$). As before, to aid interpretation of the interaction effect for any statistically significant signal, we present the association between lung function trait and air pollution stratified by genotype group.

### 2.11 Weighted genetic risk score interaction analysis

We used a weighted genetic risk score (GRS) to explore whether the combined effect of previously reported lung function signals showed an interaction with air pollution measures (i.e. whether the phenotypic effects of the SNPs were modified by exposure to air pollution). Each individual’s trait specific risk score was calculated using the effect sizes of the 279 SNPs reported in Shrine et al. 2019 (Shrine et al., 2019) on FEV_1_, FVC and FEV_1_/FVC (using the lung function reducing allele as the coded allele). Multiple regression was performed using the same model above, using the weighted GRS for each lung function trait in place of the genotype. As all three lung function traits are correlated, interaction terms (i.e. GRS × Air pollution measure) with P<0.05 were defined as statistically significant.

### 2.12 Antioxidant genes and their interaction with air pollution

Genetic variation within antioxidant genes may contribute to susceptibility of adverse effects of air pollution on respiratory health (Fuertes et al., 2020). We have provided look-ups for the most commonly evaluated antioxidant genes (for which a SNP was reported) and for SNPs evaluated in previous antioxidant-gene-air pollution interaction studies, both of which are reviewed in Fuertes et al. (Fuertes et al., 2020). A Bonferroni adjusted threshold of $P\;<\;3.85\times 10^{-3}$ (for 13 variants) was used to determine statistical significance.

## 3 Results

The association between lung function and air pollutants PM_10_, PM_2.5_ and NO_2_ in UK Biobank has previously been published in (Doiron et al., 2019), and we provide those associations in **supplementary Table 1**.

### 3.1 Genome-wide interaction analysis

**Fig. 1:**
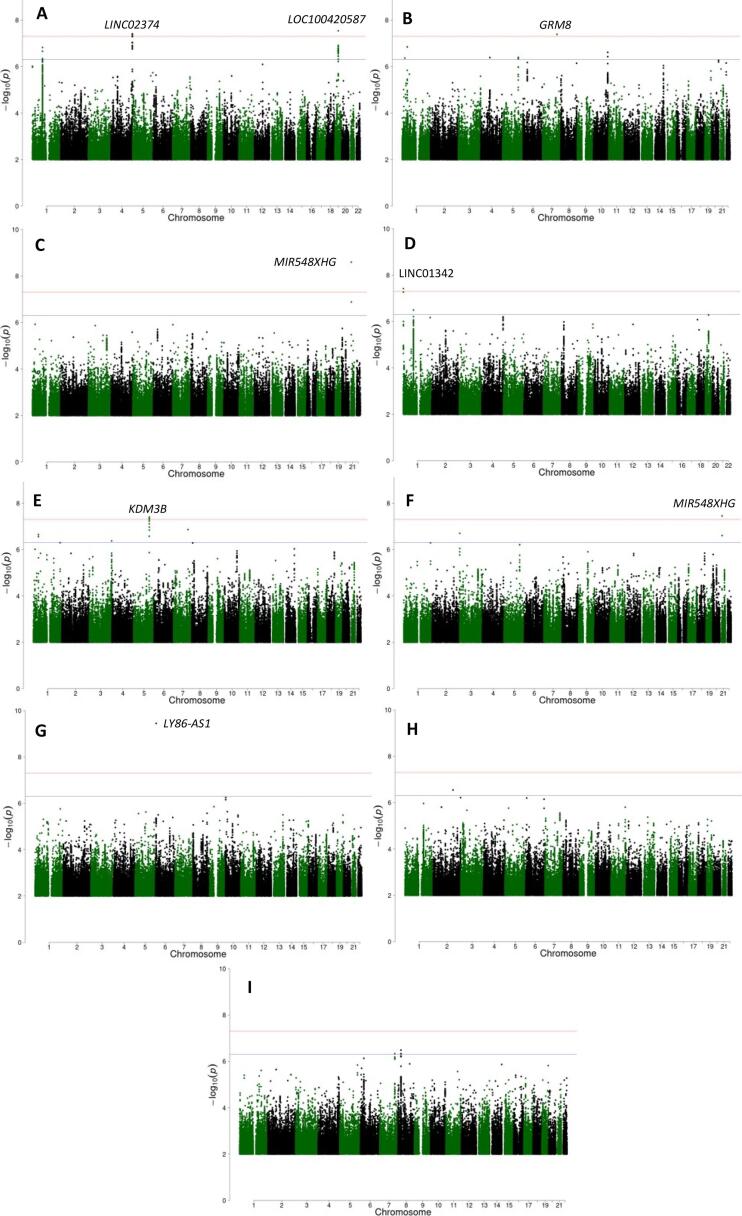
Manhattan plots for the gene-air pollution interaction GWAS (A) FEV_1_-PM_10_ (B) FEV_1_-PM_2.5_ (C) FEV_1_-NO_2_ (D) FVC-PM_10_ (E) FVC-PM_2.5_ (F) FVC-NO_2_ (G) FEV_1_/FVC-PM_10_ (H) FEV_1_/FVC-PM_2.5_ (I) FEV_1_/FVC-NO_2_. The red line represents a p-value threshold of $5\times 10^{-8}$. The blue line represents a p-value threshold of $5\times 10^{-7}$. Each genome-wide signal is annotated by nearest gene. (For interpretation of the references to colour in this figure legend, the reader is referred to the web version of this article.)

Genome-wide interaction analysis was undertaken in 277,597 European individuals from UK Biobank for air pollution variables PM_10_/PM_2.5,_ (**Supplementary Table 2**) and a total of 10,848,082 SNPs (**Supplementary Fig. 2**). For the NO_2_ analysis, there were 299,015 European individuals and 10,846,777 SNPs. Manhattan plots are presented in Fig. 1 and QQ plots in **supplementary Fig. 3**.

We identified seven signals with an interaction effect reaching genome-wide statistical significance ($P\;<\;5\times 10^{-8}$) for at least one lung function trait and air pollution variable (Table 1**, Supplementary Table 3 and Supplementary Fig. 4**). Four signals were identified for an interaction with PM_10_. There were two for FEV_1_ (in 4q35.2 [near *LINC02374*] and in 19q12 [near *LOC100420587*]), one for FVC (in 1p36.33 [near *LINC01342*]) and one for FEV_1_/FVC (in 6p25.1 [in *LY86-AS1*]). Two signals were identified for an interaction with PM_2.5_; one for FEV_1_ (in 7q31.33 [near *GRM8*]) and one for FVC (in 5q31.2 [in *KDM3B*]. One signal was identified for air pollutant NO_2_ for both lung function traits FEV_1_ and FVC (in 21q21.1 [near *MIR548XHG*]). Of the seven identified SNPs, three were common (MAF > 5%) two were low frequency, (1% < MAF < 5%) and two were rare (MAF < 1%). Conditional analysis did not identify any additional signals in each region.

**Table 1:** Seven identified genome-wide gene-air pollution interaction signals Note: CAF = Coded Allele Frequency, INFO = Imputation quality, AP = Air Pollutant, LF = Lung Function, BP = Base Position. A negative BETA (interaction effect) suggests a more deleterious effect on lung function per unit increase of air pollutant as the coded allele increases. A positive BETA (interaction effect) suggests a more protective effect. Interaction effect is per 10 μg/m^3^ increase in air pollutant NO_2_ and per 5 μg/m^3^ increase for air pollution variables PM_10_ and PM_2.5_ as the coded allele increases. Lung function effects are the product of the BETA value and the observed standard deviation of the lung function trait within the analysed sample.

LF trait	AP	SNP	CHR	BP	Coded allele	Non-Coded allele	CAF	INFO	BETA	SE	BETA effect in units of LF (ml for FEV_1_, FVC or percentage points for FEV_1_/FVC)	P	Locus
FVC	PM_10_	rs74048016	1	1,068,280	C	G	0.98	0.97	−0.139	0.025	−133.1	3.83 × 10^-8^	C1orf159 (dist = 16811), LINC01342 (dist = 4117)
FEV_1_	PM_10_	rs28666788	4	188,078,645	G	A	0.096	0.99	−0.065	0.012	−49.2	3.92 × 10^-8^	FAT1 (dist = 433635), LINC02374 (dist = 44495)
FVC	PM_2.5_	rs192415220	5	137,726,002	C	T	0.006	0.94	−0.479	0.087	−459.3	3.96 × 10^-8^	KDM3B
FEV_1_/FVC	PM_10_	rs137914543	6	6,414,006	G	GTCTC	0.06	0.83	−0.101	0.016	−0.65	3.55 × 10^-10^	LY86-AS1
FEV_1_	PM_2.5_	rs138235384	7	125,969,169	C	T	0.994	0.90	−0.466	0.085	−352.1	4.10 × 10^-8^	LOC101928283 (dist = 949794), GRM8 (dist = 109483)
FEV_1_	PM_10_	rs762101031	19	29,112,275	CAAT	C	0.96	0.79	−0.120	0.022	−90.5	2.87 × 10^-8^	LOC100420587
FEV_1_	NO_2_	rs2825255	21	20,362,376	T	C	0.83	1.00	−0.027	0.005	−20.6	2.53 × 10^-9^	MIR548XHG (dist = 230246), LINC01683 (dist = 903217)
FVC									−0.025	0.005	−24.2	3.52 × 10^-8^	

**Fig. 2:**
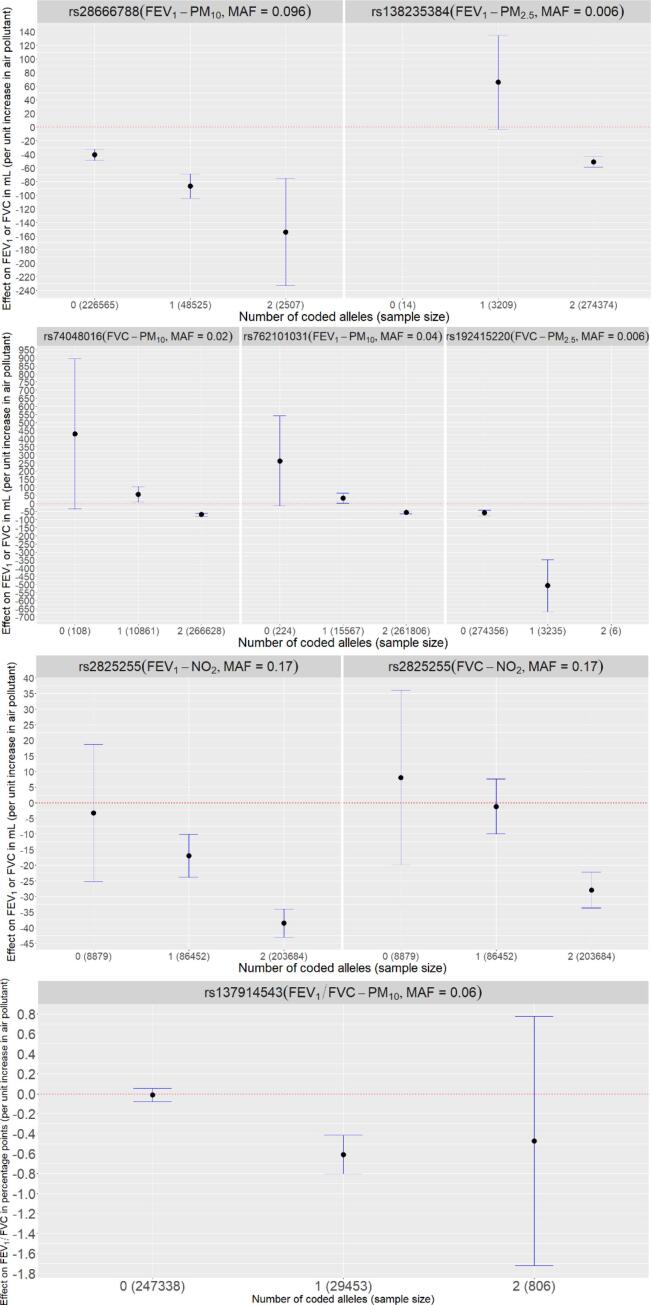
Association between lung function trait and air pollutant (effect size and confidence intervals) for the seven genome-wide signals. Note: For SNPs rs138235384 and rs192415220 the effect size for 0 copies and 2 copies of the effect allele respectively are not presented due to the low minor allele frequency and small sample size. Effect sizes will not be exactly consistent with Table 1 due to rounding error when converting from dosage to direct genotypes. Units are per 5 µg/m^3^ for PM_10_ / PM_2.5_ and per 10 µg/m^3^ for NO_2_.

To aid with the interpretation of statistically significant interaction effects, we have presented the association between air pollution and lung function stratified by genotype group (number of copies of coded allele) for each of the seven genome-wide interaction signals (Fig. 2**)** and interaction plots of predicted lung function against air pollution for each genotype group (**Supplementary Fig. 5)**. In some instances, statistically significant association between lung function and air pollutant is observed in all genotype groups. For others, the association only reaches statistical significance for certain genotype groups.

Signals were deemed suggestively statistically significant using the same signal selection procedure with a threshold of $P\;<\;5\times 10^{-7}$ (**Supplementary Table 4 and Supplementary Fig. 6**). Region plots after conditional analysis suggested only one signal per 2 Mb region. Ten suggestive signals were identified that were independent of the seven genome-wide significant signals, all were either intergenic or mapped to the intronic region of the mapped gene. Eight were represented by common SNPs, and two by low frequency SNPs.

### 3.2 Credible sets and causal genes

To identify the gene (or genes) via which the SNPs (for genome-wide and suggestively significant signals) might be exerting their effects on lung function, we used a Bayesian method to refine our signal to define the 95% credible set of causal SNPs (assuming the causal SNP was included in the analysis, **Supplementary Table 5**). We then investigated whether credible set and sentinel SNPs were associated with changes in gene expression in GTEx, Blood eQTL and Open Target Genetics databases (**Supplementary Table 6**). Genome-wide significant signal, rs74048016, whose C allele had a larger deleterious effect on lung function as the measurement of PM_10_ increased, was associated with decreased expression of *HES4* and increased expression of *C1orf159* and *RP11-465B22.3* in blood. However, there was no statistical support (using COLOC (Giambartolomei et al., 2014) and the eQTLgen database (Võsa et al., 2021) that the interaction signal and gene expression association signals originated from the same SNPs. Credible set SNPs for suggestive signals rs769937512, rs111552599, rs139556451, rs200460259 and rs10082259 were associated in various tissues for genes *AL445991.1, FRAS1*, *PNMA2*/*DPYSL*, *MUC4*/*MUC20* and *UROD* respectively (**Supplementary Table 6**). These signals did not colocalise, suggesting again that the observed gene expression and interaction signals were not driven by the same SNPs. There was no association with protein expression and no overlap with regions that had strong evidence for interaction with gene promoters.

### 3.3 Association with other phenotypes

The sentinel SNPs for the 17 genome-wide and suggestively significant signals were queried in PhenoScanner and Open Targets Genetics resources (**Supplementary Table 7**), to explore their association with related phenotypes e.g. asthma that might support a causal interpretation. Five signals were found to be associated with at least one trait at $P\;<\;1\times 10^{-3}$, three genome-wide signals (rs28666788, rs192415220 and rs138235384) and two suggestive signals (rs10082259/rs6661026 and rs769937512), but none of the associations had reached genome-wide significance ($P\;<\;5\times 10^{-8})$. For the genome-wide signals rs28666788, rs192415220 and rs138235384 the strongest associations (at $P\;<\;5\times 10^{-6}$) were with alcohol consumption, self-reported cervical polyps and sexual dysfunction respectively.

### 3.4 Tissue-specificity of interaction signals

When looking for evidence that the interaction signals were over-represented in tissue-specific functionally active regions of the genome (DNase I hypersensitive sites (DHS) indicative of open chromatin) using GARFIELD or responsible for chromatin effects using DeepSEA, only SNPs showing SNP-NO_2_ interaction effects on lung function phenotype FVC were enriched in various tissues including fetal lung, using a threshold of $P\;<\;5\times 10^{-5}$ to select contributing SNPs (**Supplementary Fig. 7 and Supplementary Table 8**).

### 3.5 Sensitivity analyses

#### 3.5.1 Effects of Socio-Economic status

When adjusting for socio-economic status variables educational status and income status, sample sizes were reduced to 259,130 and 240,202 for the NO_2_ and PM_10_/PM_2.5_ analyses respectively. Effect sizes were largely consistent with the primary analysis with minimal reductions in effect size for rs74048016 and rs192415220 (**Supplementary Table 9 and Supplementary Fig. 8**), suggesting that the interactions identified were not due to confounding by SES factors. Interaction effects were generally larger in magnitude (but not significantly due to overlapping confidence intervals) for those in the lower educational group (**Supplementary Fig. 9**). When stratifying by income group (**Supplementary Fig. 10**), overlapping confidence intervals again suggested no significant effect of income status on air pollution and lung function association across genotype groups. A slight inverse correlation between magnitude of interaction effect and income group was observed for rs2825255 for both lung function traits (higher income group, smaller interaction effect magnitude) with a positive correlation observed for rs762101031 (higher income group, larger interaction effect magnitude).

#### 3.5.2 Exposure misspecification

There was very little effect on effect estimates and statistical significance for identified genome-wide interaction signals when modelling a non-linear effect of air pollution on lung function (**Supplementary Table 10**).

### 3.6 Lung function associated signals

**Fig. 3:**
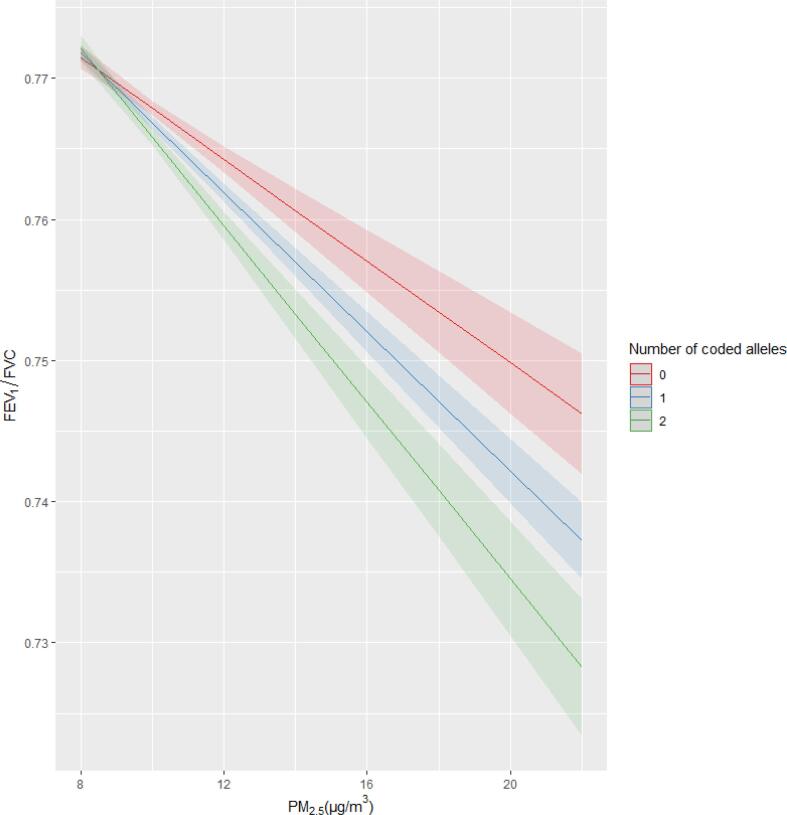
Interaction plot of FEV_1_/FVC predicted values against PM_2.5_ values across genotype groups (with coded allele G) for the previously identified lung function signal rs10841302.

To determine whether any signals previously shown to be associated with lung function produced an interaction effect with air pollution variables, we performed a look up of the 304 variants (279 lung function signals from Shrine et al. (Shrine et al., 2019) and 24 COPD signals from Sakornsakolpat et al. (Sakornsakolpat et al., 2019)) in our genome-wide analysis. Of the 304 signals, one signal, rs10841302, near *AEBP2*, for which the G allele is associated with lower values of FEV_1_/FVC, met a Bonferroni threshold of P < $1.6\times 10^{-4}$ for an interaction with PM_2.5_ for FEV_1_/FVC (interaction β: -0.0569; 95% CI: − 0.0826, -0.0312; interaction P = 9.65x10^-6^) (**Supplementary Table 11)**, suggesting a larger deleterious effect of PM_2.5_ on FEV_1_/FVC as copies of the G allele increased (Fig. 3). This is equivalent to an FEV_1_/FVC effect of −0.363 percentage points (CI: − 0.529, -0.200) per 5μ g/m^3^ increase in PM_2.5_. The interaction can also be interpreted by air pollution and lung function association stratified by genotype group. For genotype groups CC, CG and GG for SNP rs10841302, a 5μ g/m^3^ increase in PM_2.5_ resulted in a reduction of FEV_1_/FVC by 0.16 (95% CI: 0.13–0.19;  $P\;=\;1.21\times 10^{-22}$), 0.17 (95% CI: 0.14–0.200; $P\;=\;5.52\times 10^{-39}$) and 0.28 (95% CI: 0.24–0.32;  $P\;=\;2.43\times 10^{-43}$) standard deviations. This equates to direct FEV_1_/FVC effects of 1.024 (95% CI: 0.83–1.22.), 1.08 (95% CI: 0.90–1.28.) and 1.79 (95% CI: 1.54–2.04) percentage points respectively per 5μ g/m^3^ of PM_2.5_.

We tested the interaction between a weighted GRS for lung function (based on the effect sizes of 279 lung function signals reported in Shrine et al. (Shrine et al., 2019) and each air pollution measure on FEV_1_, FVC and FEV_1_/FVC (**Supplementary Table 12**). None of the interaction effects were statistically significant (all P > 0.05).

### 3.7 Antioxidant genes and their interaction with air pollution

We performed a look up of the 13 variants corresponding to seven commonly evaluated antioxidant genes and/or those analysed in previous studies of antioxidant gene-air pollution interaction analyses, as reviewed by Fuertes et al. (Fuertes et al., 2020) (**Supplementary Table 13**). None of the SNPs reached the Bonferroni significant adjusted threshold used to determine statistical significance ( $P\;<\;3.85\times 10^{-3}$). One SNP, rs1001179 in *CAT* approached this threshold (P = 0.009) for an interaction with NO_2_ for FEV_1_/FVC.

## 4 Discussion

We carried out the largest genome-wide gene-air pollution interaction study of lung function and identified seven genome-wide statistically significant signals, as well as identifying a small interaction with air pollution for one previously identified lung function signal. Independent replication is required to confirm these results. There were no interactions detected between air pollution and a weighted genetic risk score for lung function (using previously identified lung function signals), nor with seven commonly evaluated antioxidant genes. Further, we did not see convincing evidence of effect modification by social class.

For the signals identified, ascribing the biological mechanisms proves a challenge and further biological studies of gene function for those implicated are needed. For genome-wide SNP rs74048016, as the number of copies of the coded allele increases the effect of air pollutant PM_10_ on FVC becomes more negative, suggesting that those with two copies of the effect allele are at increased susceptibility of air pollution effects. The coded allele is associated with decreased expression of *HES4* and increased expression of *C1orf159* in blood in Open Targets Genetics. The signals for genome-wide association and gene expression signals did not colocalise (there was insufficient evidence of a shared causal variant between the two analyses) in this genomic region (using data from eQTLgen). Expression of *HES4 (*hes family bHLH transcription factor 4) has been implicated in poor outcomes for patients with Triple Negative Breast Cancer (TNBC) (Stoeck et al., 2014) and both *HES4* and *C1orf159* (chromosome 1 open reading frame 159*)* have been implicated via functional annotation (nearest gene) of other genome-wide significant loci for several traits and diseases, including peak expiratory flow (PEF) (Ghoussaini et al., 2021, Neale Lab, 2021). There is also evidence of colocalisation between gene expression and genome-wide analyses for these genes in certain tissues for height phenotypes (standing and sitting) (Ghoussaini et al., 2021, Neale Lab, 2021).

We identified a further ten signals (independent of the primary genome-wide signals) at suggestive statistical significance, which would be important to take forward in future replication analyses. Genes implicated include *PNMA2*, *DPYSL2* and *BNIP3L*, all via functional annotation of other genome-wide significant loci for height, and additionally for educational attainment phenotypes (Kichaev et al., 2019, Lee et al., 2018). There was however no attenuation of suggestive signal rs139556451 (which implicated the aforementioned genes in our analysis) when re-analysing with adjustment for education and income status (in the subset for which this data was available). *BNIP3L* expression has also been linked with lung cancer (Sun, et al., 2004). Additionally, gene *FRAS1* identified by eQTL associations for SNPs in the rs111552599 suggestive signal credible set has been implicated by other genome-wide signals for lung function, specifically for trait FEV_1_/FVC (Kichaev et al., 2019, Shrine, 2019) and mutations in *FRAS1* have been observed amongst individuals with Fraser syndrome, which can cause airway abnormalities (Pitera et al., 2008, van Haelst et al., 2007). *MUC4* (identified by credible set eQTL associations for rs200460259), which encodes airway mucins (Copin et al., 2000) is associated with severity of lung disease in cystic fibrosis (through functional annotation of another genome-wide signal) (Corvol et al., 2015) and risk of lung cancer (association with variants in the gene) (Zhang et al., 2013). We were however unable to determine whether the association signal for the genes described here were driven by the same causal variant as the interaction signal.

We identified an interaction effect between SNP rs10841302 (a previously identified lung function signal associated with FEV_1_/FVC) and PM_2.5_ for lung function trait FEV_1_/FVC. Previous work has shown that the rs10841302 G-allele is associated with a deleterious effect on FEV_1_/FVC. We found that this deleterious effect increased in magnitude as the exposure to PM_2.5_ increased. A causative gene for the association between rs10841302 and lung function has not been determined. The SNP is near *AEBP2* (AE Binding Protein 2), a transcriptional repressor with a possible contribution to histone methylation and the G allele is associated with increased expression of both *RP11-405A12.2* (in pancreas and subcutaneous adipose tissues) and *RP11-664H17.1* (in pancreas and tibial nerve tissues) in GTEx (GTEx Consortium, 2013). There was no evidence of an interaction between air pollution measures and a combined effect from all previously identified lung function signals represented by a genetic risk score.

A particular strength of this study is the discovery sample size available for the interaction analysis, despite resulting in relatively few findings. This is likely indicative of the fact that several environmental and other exposures are at interplay across an individual’s life course, and these are not addressed in our analysis. Moreover, interactions are challenging to identify due to the requirement of much larger sample sizes than GWAS efforts exploring the marginal effects of genetic variants (Thomas, 2010). This strength is however unfortunately a contributor to its biggest limitation, which is identifying suitable independent datasets of sufficient sample size with lung function data in European ancestry populations to replicate discovery interaction signals. We calculated that sample sizes to replicate three of our novel genome-wide interaction signals when considering the reported interaction effect, main genetic effect and air pollution variable effect (chosen from each MAF frequency group of common, low frequency and rare), signals rs28666788 (MAF = 10%), rs74048016 (MAF = 2%) and rs192415220 (MAF = 0.6%) would be ∼ 72 k, ∼ 71 k and ∼ 66 k respectively to detect the effect at 80% power. However, these sample sizes are indeed sensitive to any observed error in interaction effect estimates, such that when using lower and upper confidence interval effect estimates, sample sizes required could range from ∼ 35 k to ∼ 194 k.

The discovery of gene-air pollution interactions which affect lung function susceptibility is limited, likely due to the aforementioned difficulty in identifying suitable sample sizes to provide adequate power for replication studies, which is a limitation of our present analysis. Previous genome-wide interaction studies are either attributed to related phenotypes, such as asthma (Gref et al., 2017) or have focussed on candidate genes, such as those with a role in oxidative stress, where conclusions drawn are often inconsistent with respect to direction of effect or presence of interaction (Minelli et al., 2011, Romieu et al., 2010). Previous studies of interactions between genes and smoking behaviour, the largest risk factor for poor lung function and COPD, have also been largely unsuccessful in identifying interaction signals. This has been of interest as not all smokers develop restrictive lung problems. Candidate gene-smoking interactions have been identified, however utilising small sample sizes with absence of replication (Sadeghnejad et al., 2007, Hunninghake et al., 2009, He et al., 2004) and none of the previously identified lung function signals produced an interaction with smoking behaviour (Shrine et al., 2019). Genome-wide interaction analysis efforts have also been considered for lung function (Hancock et al., 2012) however with little success, and although a recent study of gene-smoking interaction effects for COPD found a genome-wide significant interaction at *15q25.1* (Kim et al., 2020), this is likely driven by the strong association between this locus and smoking behaviour (Thorgeirsson et al., 2010, Liu et al., 2010, The Tobacco and Genetics Consortium, 2010). There has however been some evidence of interaction between smoking behaviour and genetic risk scores, when combining the effects of SNPs associated with lung function (Aschard et al., 2017, Shrine, 2019). To the best of our knowledge, no genome-wide significant smoking interaction signals for lung function have been identified, highlighting the impact of identifying novel genome-wide gene-air pollution interaction signals.

Should the interaction effects be replicated in future analyses, the magnitude of effects observed here suggest potential for clinically relevant impacts on those with certain genotypes. Results (Table 1**,** Fig. 2) are expressed per 5 µg/m^3^ for air pollutants PM_10_ and PM_2.5_ and per 10 µg/m^3^ for NO_2_. For context, average annual concentrations of PM_10_ in 2018 were 14.7 µg/m^3^ in 2018 at urban background air quality monitoring sites (likely to represent where most of the UK population live) (GOV.UK, 2021). Corresponding concentrations for PM_2.5_ and NO_2_ was 10.0 µg/m^3^ and 20.1 µg/m^3^ respectively. Taking genome-wide signal rs28666788 as an example, (with coded allele G frequency of 0.096), effects on FEV_1_ per 5 µg/m^3^ increase in PM_10_ were statistically significant for all genotype groups. For those with zero, one and two copies of the effect allele, lung function effects of approximately −40 ml, −87.5 ml and −150 ml were observed per 5 µg/m^3^ PM_10_ respectively (Fig. 2). Therefore, when subjected to the average concentrations of 14.7 µg/m^3^ of PM_10_, this equates to respective reductions of approximately 118 ml, 260 ml and 440 ml. Average declines in FEV_1_ per year could be up to 46 ml for individuals aged 30 onwards (Quanjer et al., 2012), so these effects are approximately equivalent to nine years of normal loss of lung function for those with two copies of the coded allele (4 and 7 more than those with one and zero copies respectively). For other SNPs, such as rs2825255, with coded allele (T) frequency of 0.83, association between lung function and air pollutant is observed for certain genotype groups. Using the average NO_2_ measure, those with one and two copies of the effect allele could be subject to reductions in FEV_1_ of approximately 35 ml and 75 ml (approximately equivalent to 0.75 and 1.5 years of normal lung function decline respectively), as opposed to those with zero copies, where there was no observed statistically significant effect of air pollutant on FEV_1_ (confidence interval overlaps 0).

There were approximately 40,000 individuals with clean lung function data with missing data for education and income status. We expect that those with higher SES and higher income are more likely to have complete data thus the data is not missing at random. We did not carry out imputation as it is difficult to know which might introduce more bias, imputation or exclusion and thus carried out a complete-case analysis. Further studies are required in this respect. Previous studies have reported modification of air pollution effects on lung function when considering SES (Doiron et al., 2017, Wheeler and Ben-Shlomo, 2005, Forastiere et al., 2007, Doiron et al., 2019) possibly due to differences in housing conditions, indoor air quality, nutrition and occupation (Forastiere et al., 2007). Adjusting for SES and presenting interaction effects across educational and income groups did not produce a notable modification of interaction effects in our analyses, suggesting that observed differences in the effect of air pollution across genotype groups are not mediated or confounded by socio-economic status.

There are other limitations with this study. We only had air pollution data at baseline with some limitations in the availability and did not have follow-up data. An analysis of a German cohort of 601 elderly women (mainly non-smokers) with three follow-ups from 1985 to 2013 suggested that changes in air pollution over time was associated with improvements in lung function, modified by genetic factors (Hüls et al., 2019). In addition, there are limitations with the ESCAPE models (Eeftens et al., 2012, Beelen et al., 2013). Exposure estimates are based on place of residence so will not capture variability in exposure related to work and leisure activities outside the home, which may have led to exposure misclassification bias making it harder to detect effects. Furthermore, it must be noted that our analysis includes imputed genetic dosages alongside directly genotyped data and we only considered an additive genetic model for our analysis. Previous studies for certain antioxidant gene SNPs such as rs1695 in *GSTP1* have also considered the suitability of alternative genetic models (Wang et al., 2019, Song et al., 2016).

In conclusion, we have identified genetic variants whose effect on lung function is dependent on air pollution exposure levels. This could help identify high-risk genetic subgroups whose lung function could be more susceptible to the effects of outdoor air pollution. While this is the largest study of this type to date, we highlight the need for replication in independent datasets with recorded lung function, for which availability is currently limited. We hope that future replication and further biological studies of gene function will help to establish the genes and biological pathways involved.

## CRediT authorship contribution statement

**Carl A. Melbourne:** Conceptualization, Methodology, Software, Validation, Investigation, Formal analysis, Data curation, Writing – original draft, Writing – review & editing, Visualization. **A. Mesut Erzurumluoglu:** Conceptualization, Methodology, Software, Writing – review & editing. **Nick Shrine:** Software, Resources, Writing – review & editing. **Jing Chen:** Software, Resources, Writing – review & editing. **Martin D. Tobin:** Conceptualization, Resources, Supervision, Funding acquisition, Writing – review & editing, Project administration. **Anna L. Hansell:** Conceptualization, Investigation, Resources, Supervision, Project administration, Funding acquisition, Writing – review & editing. **Louise V. Wain:** Conceptualization, Project administration, Supervision, Funding acquisition, Writing – review & editing.

## Declaration of Competing Interest

The authors declare that they have no known competing financial interests or personal relationships that could have appeared to influence the work reported in this paper.
