# Supplementary material for: Genome-wide gene-air pollution interaction analysis of lung function in 300,000 individuals
Source: Environ Int. 2022 Jan 15;159:107041. doi: 10.1016/j.envint.2021.107041 (PMC8739564; doi:10.1016/j.envint.2021.107041)

# Supplementary figures

**Table of Contents**

[Supplementary figures 1](#_Toc86067963)

[Figure S1. Histograms of air pollution variables PM_10_, PM_2.5_ and NO_2_ 2](#_Toc86067964)

[Figure S2. SNP quality control flow chart 3](#_Toc86067965)

[Figure S3. QQ plots for GWAS (with genomic inflation factor, λ) 4](#_Toc86067966)

[Figure S4. Region plots for genome-wide signals 5](#_Toc86067967)

[Figure S5. Interaction plots for genome-wide signals 9](#_Toc86067968)

[Figure S6. Region plots for suggestive signals 13](#_Toc86067969)

[Figure S7. GARFIELD analysis (for SNP-NO_2_ interaction analysis and FVC phenotype) 23](#_Toc86067970)

[Figure S8. Effect of SES adjustment on genome-wide signals 24](#_Toc86067971)

[Figure S9. Interaction effects stratified by education group 25](#_Toc86067972)

[Figure S10. Interaction effects stratified by income group 27](#_Toc86067973)

## Figure S1. Histograms of air pollution variables PM_10_, PM_2.5_ and NO_2_


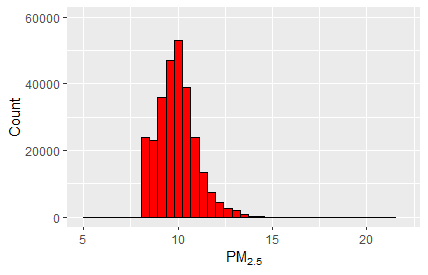

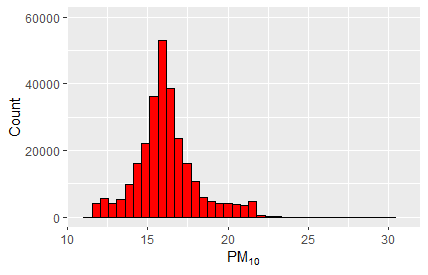

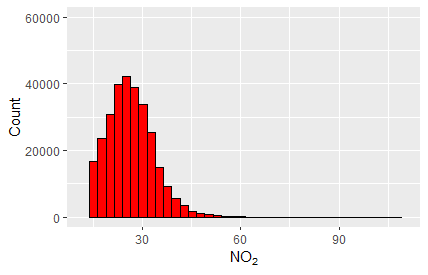


**Mean = 16.2**

**Median = 16.0**

**Variance = 3.6**

**Mean = 26.1**

**Median = 25.6**

**Variance = 54.6**

**Mean = 9.9**

**Median = 9.9**

**Variance = 1.1**

## Figure S2. SNP quality control flow chart

93,095,623 SNPs

92,457,702 SNPs

637,921 multiallelic SNPs removed

63,388,772 SNPs with low imputation quality removed

10,848,082 SNPs analysed

10,846,777 SNPs analysed

PM_10_/PM_2.5_ analyses

NO_2_ analysis

18,222,153 SNPs MAF < 0.5% removed

18,220,848 SNPs MAF < 0.5% removed

## Figure S3. QQ plots for GWAS (with genomic inflation factor, λ)

**NO_2_**

**PM_2.5_**

**PM_10_**


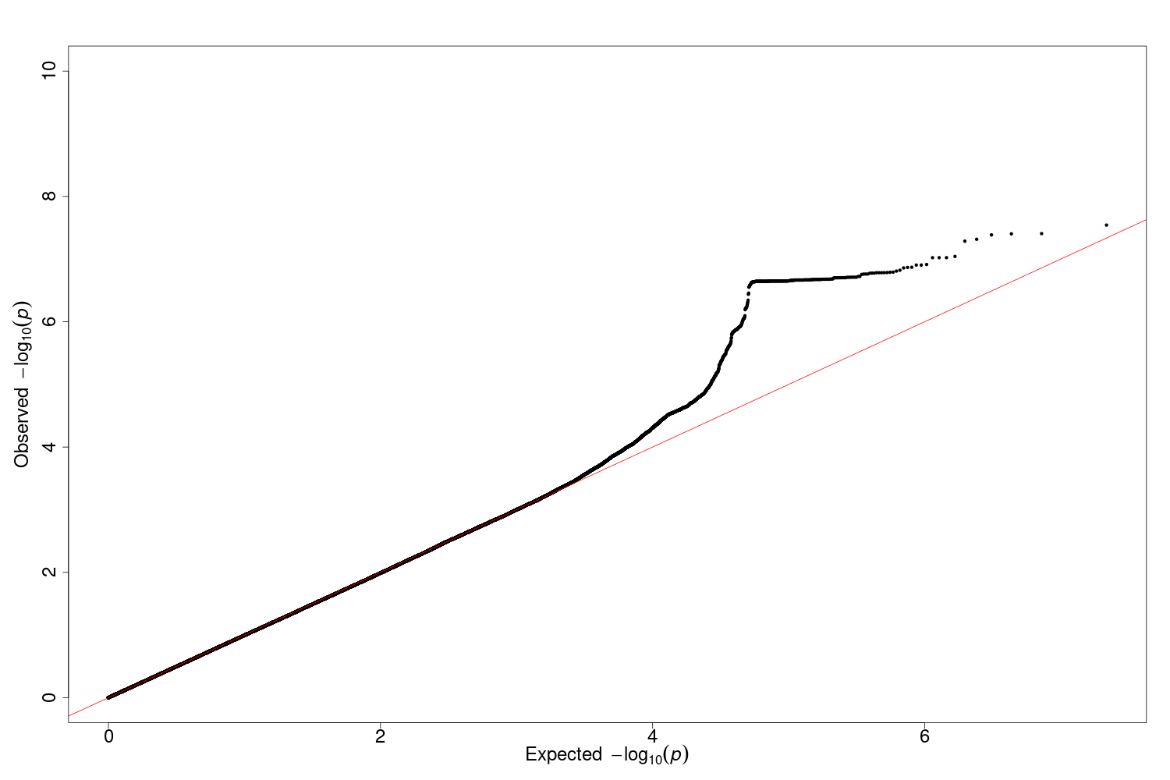

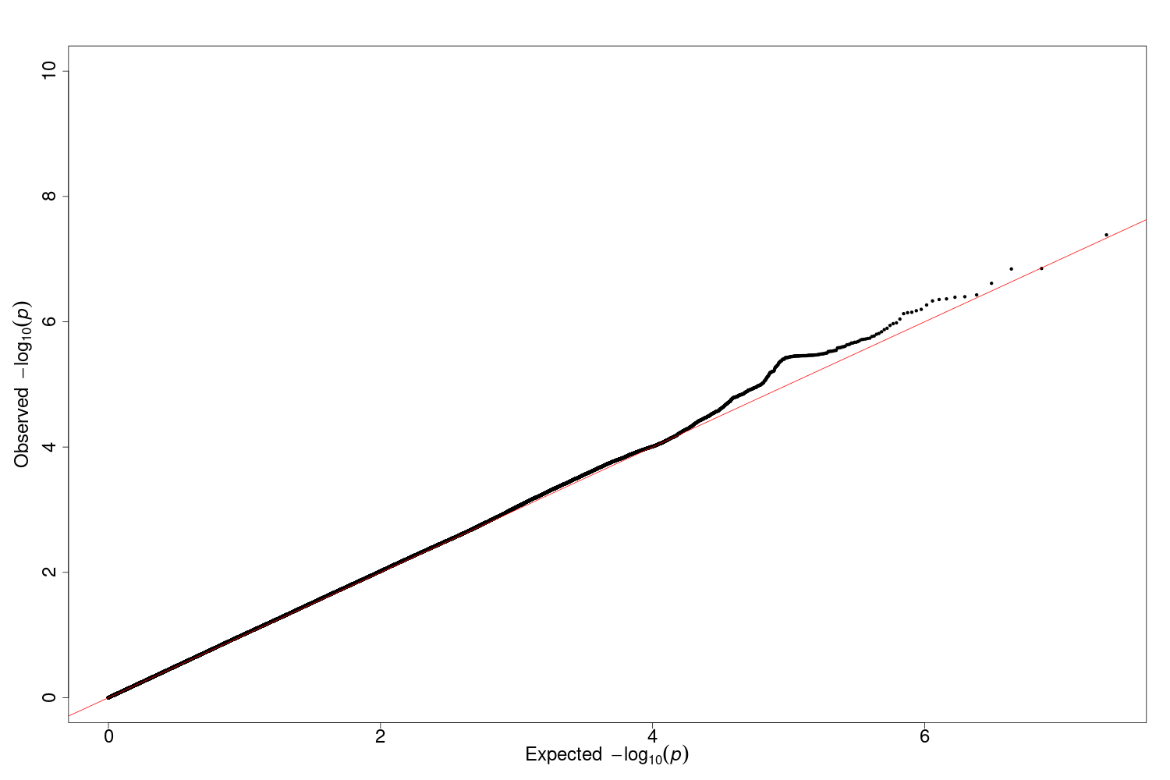

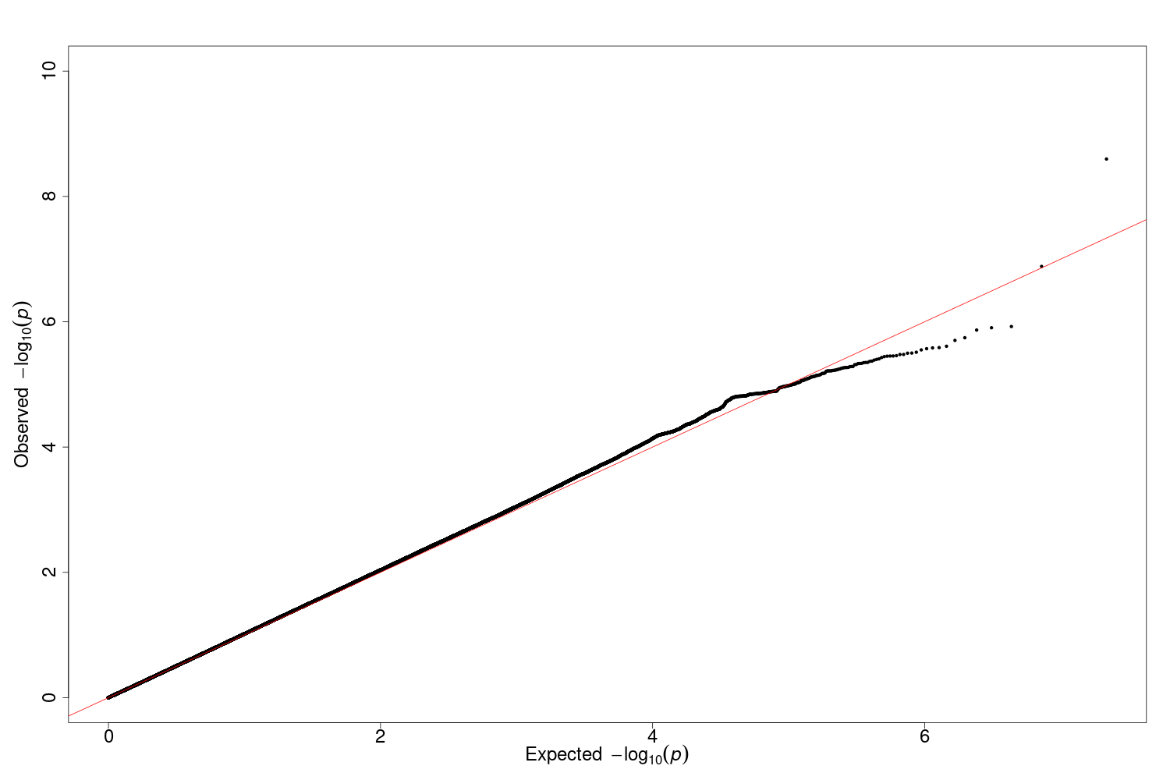

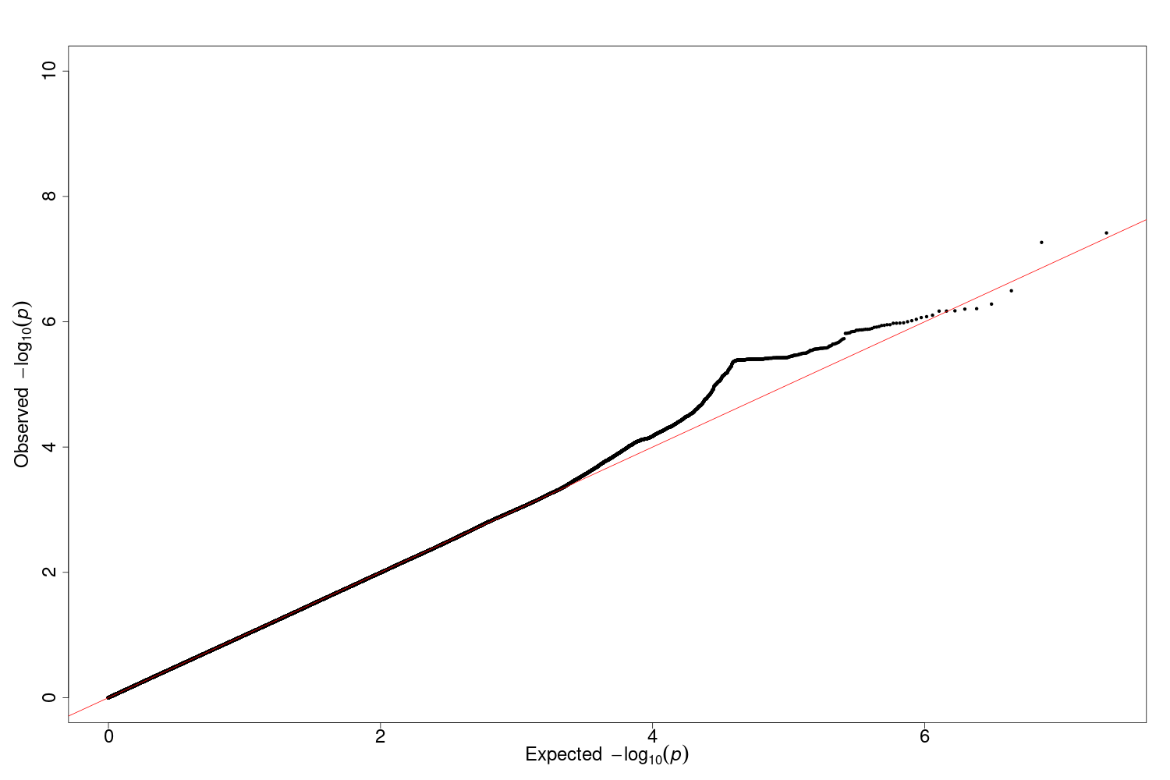

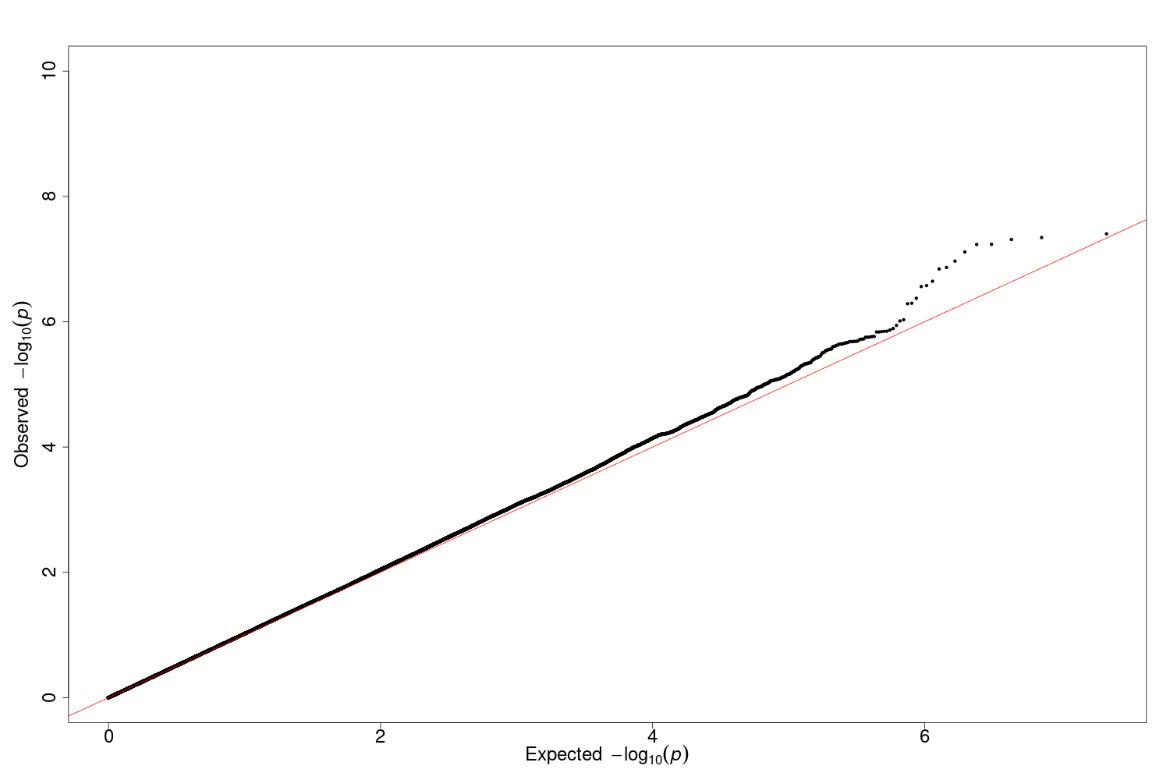

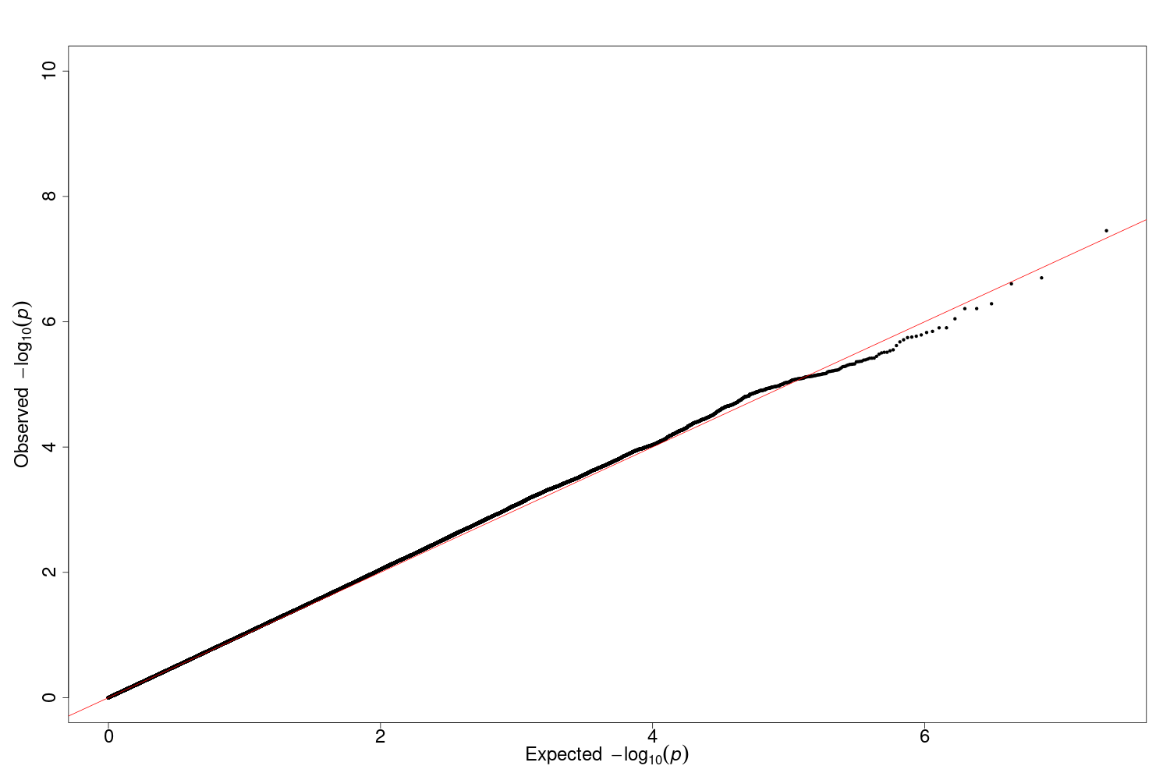

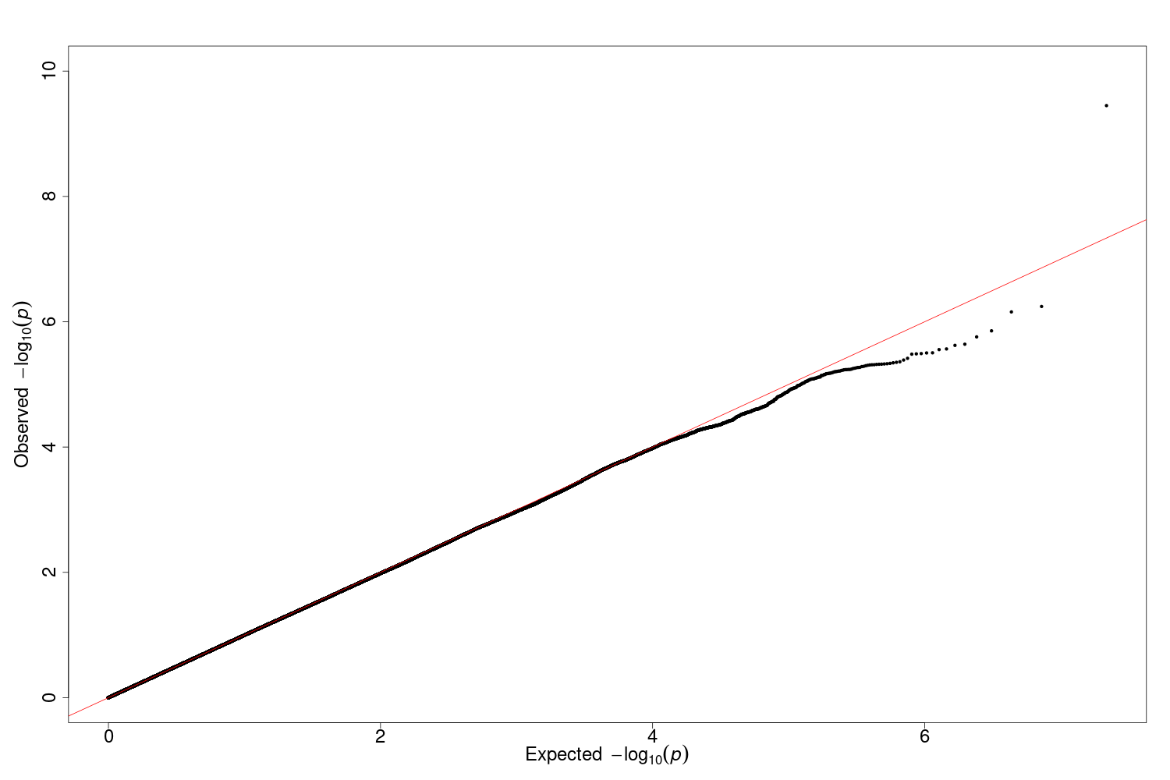

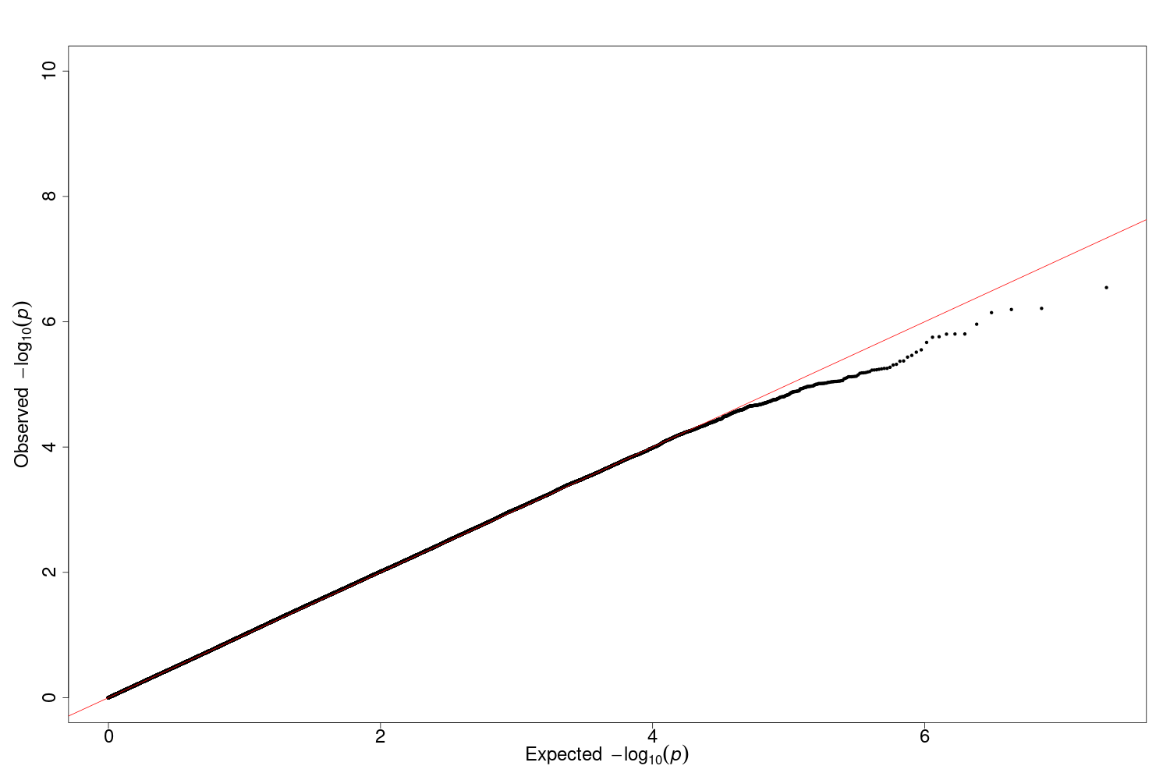

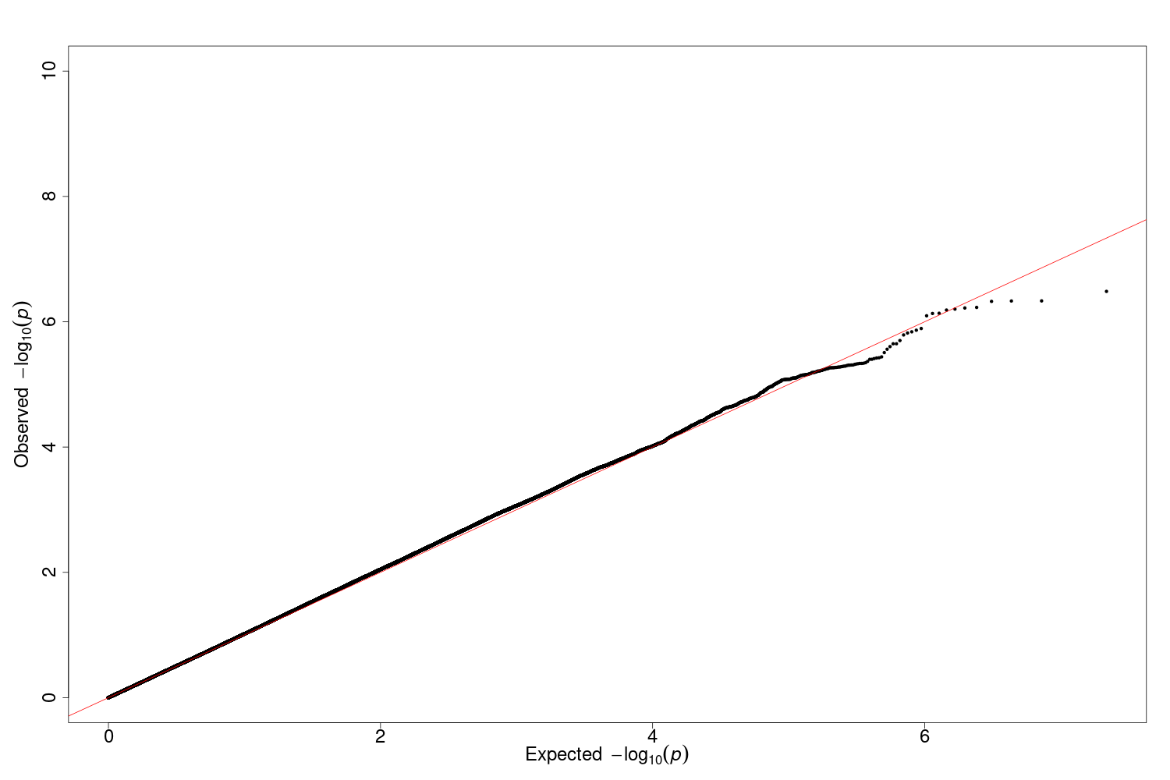


λ = 1.018

λ = 1.016

λ = 0.989

λ = 1.002

λ = 1.030

λ = 1.018

λ = 1.007

**FEV_1_**

**FVC**

λ = 0.996

**FEV_1_/FVC**

λ = 1.020

Figure S4. Region plots for genome-wide signals (A) rs74048016 (B) rs28666788 (C) rs192415220 (D) rs137914543 (E) rs138235384 (F) rs762101031 (G) rs2825255 (for FEV_1_ phenotype association) (H) rs2825255 (for FVC phenotype association)

1.
2.


1.
2. ****

1.


Figure S5. Interaction plots for genome-wide signals (A) rs74048016 (B) rs28666788 (C) rs192415220 (D) rs137914543 (E) rs138235384 (F) rs762101031 (G) rs2825255 (for FEV_1_ phenotype association) (H) rs2825255 (for FVC phenotype association)


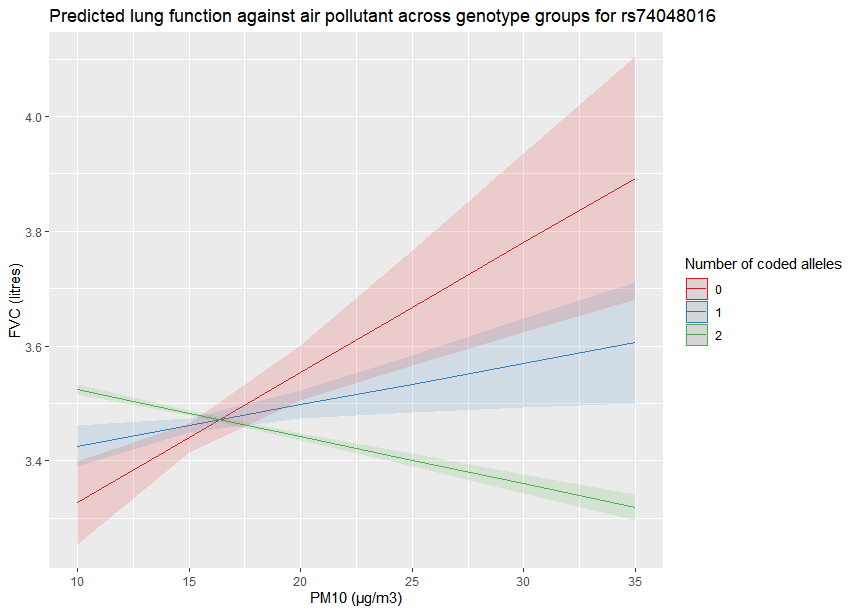


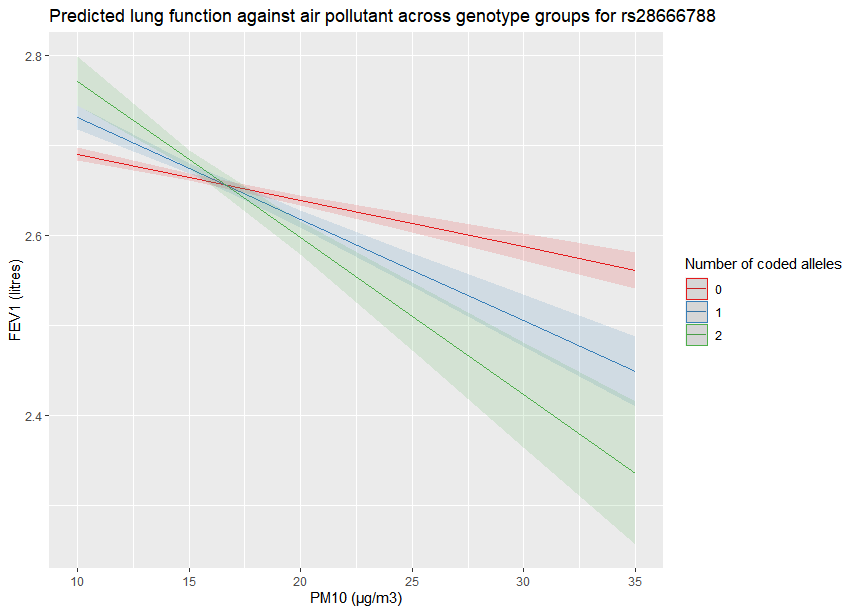


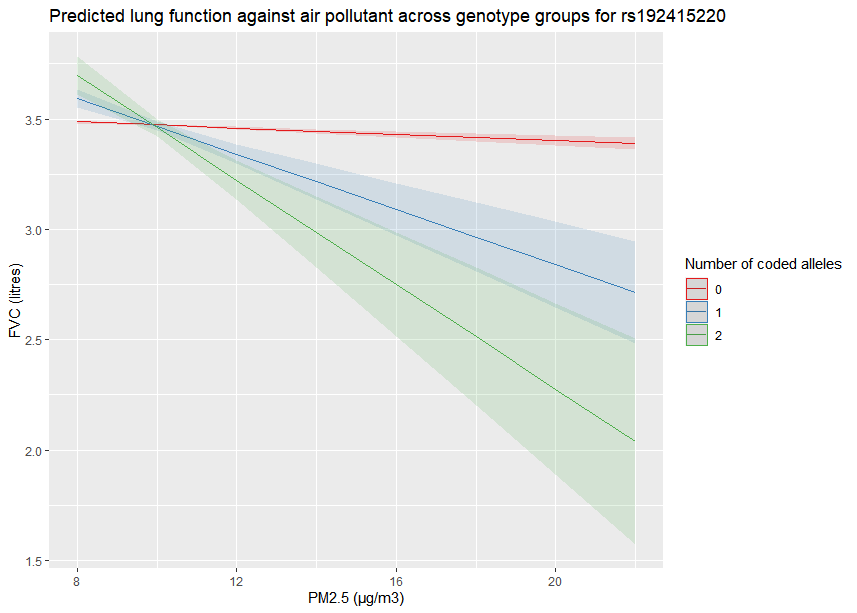


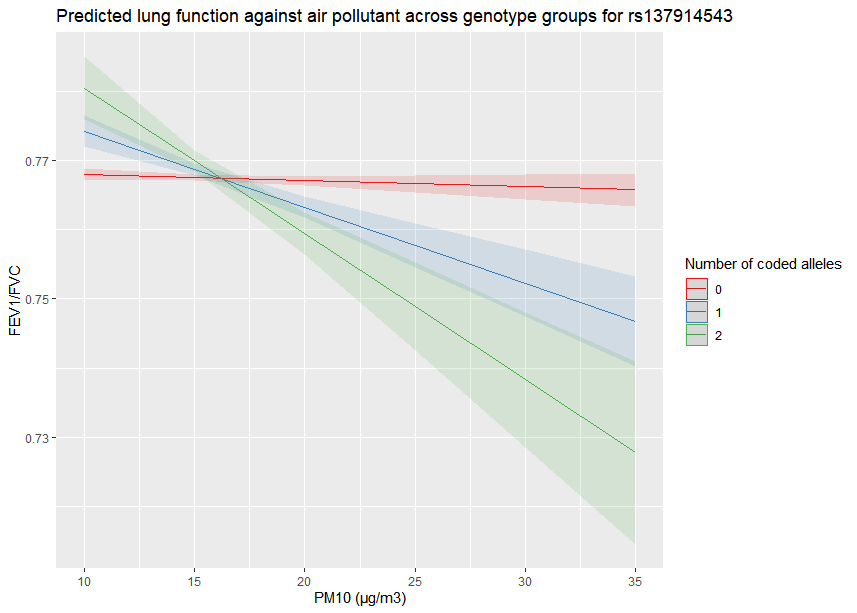


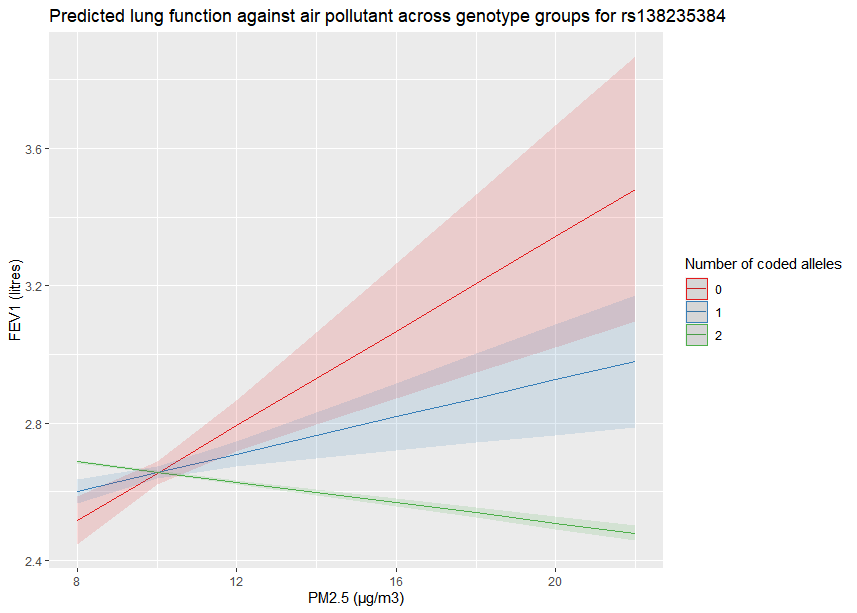


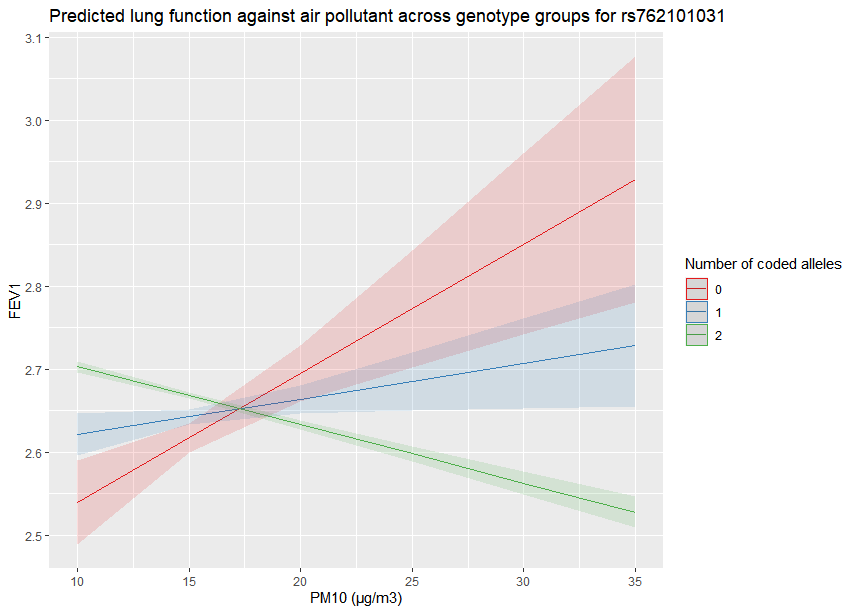


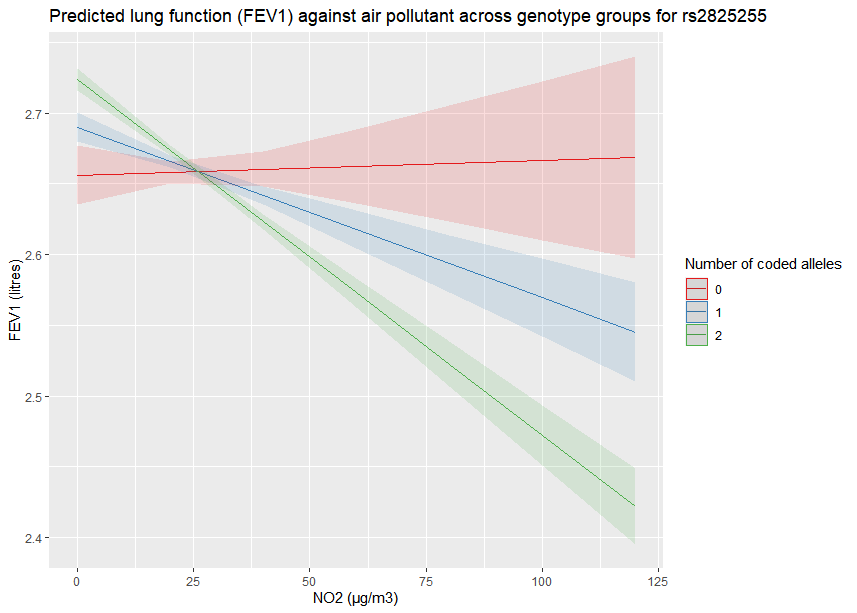


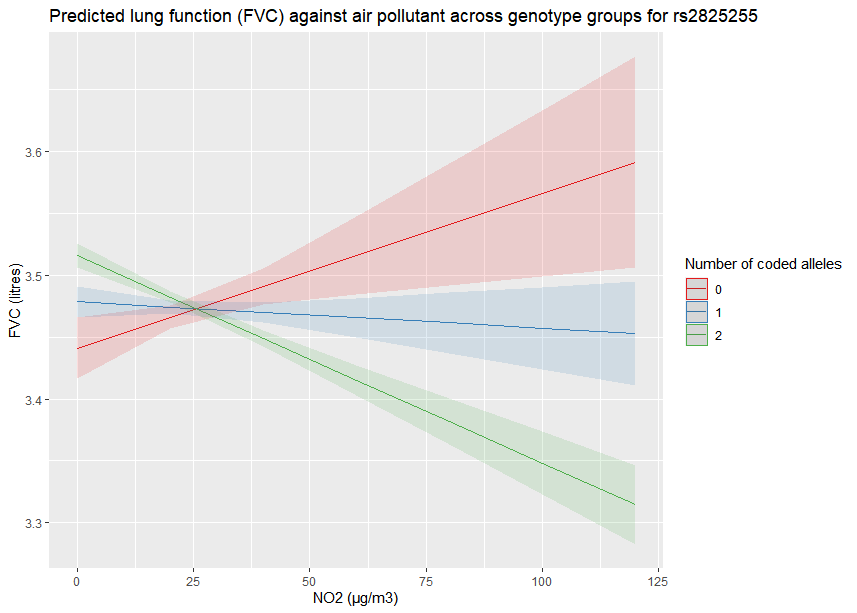


Figure S6. Region plots for suggestive signals (A) rs140250292 (B) rs35380252 (C) rs6661026 (D) rs10082259 (E) rs769937512 (FEV_1_, PM_10_) (F) rs769937512 (FVC, PM_10_) (G) rs11677115 (H) 3:322596_CCACA_C (I) rs200460259 (J) rs111552599 (K) rs28665554 (L) rs189103140 (M) rs192415220 (N) rs138235384 (O) rs73163133 (P) rs139556451 (Q) rs7098338 (R) 19:29108143_GA_G (S) rs111676952 (FEV_1_, NO_2_) (T) rs111676952 (FVC, NO_2_)

1.

1.

## Figure S7. GARFIELD analysis (for SNP-NO_2_ interaction analysis and FVC phenotype)

**
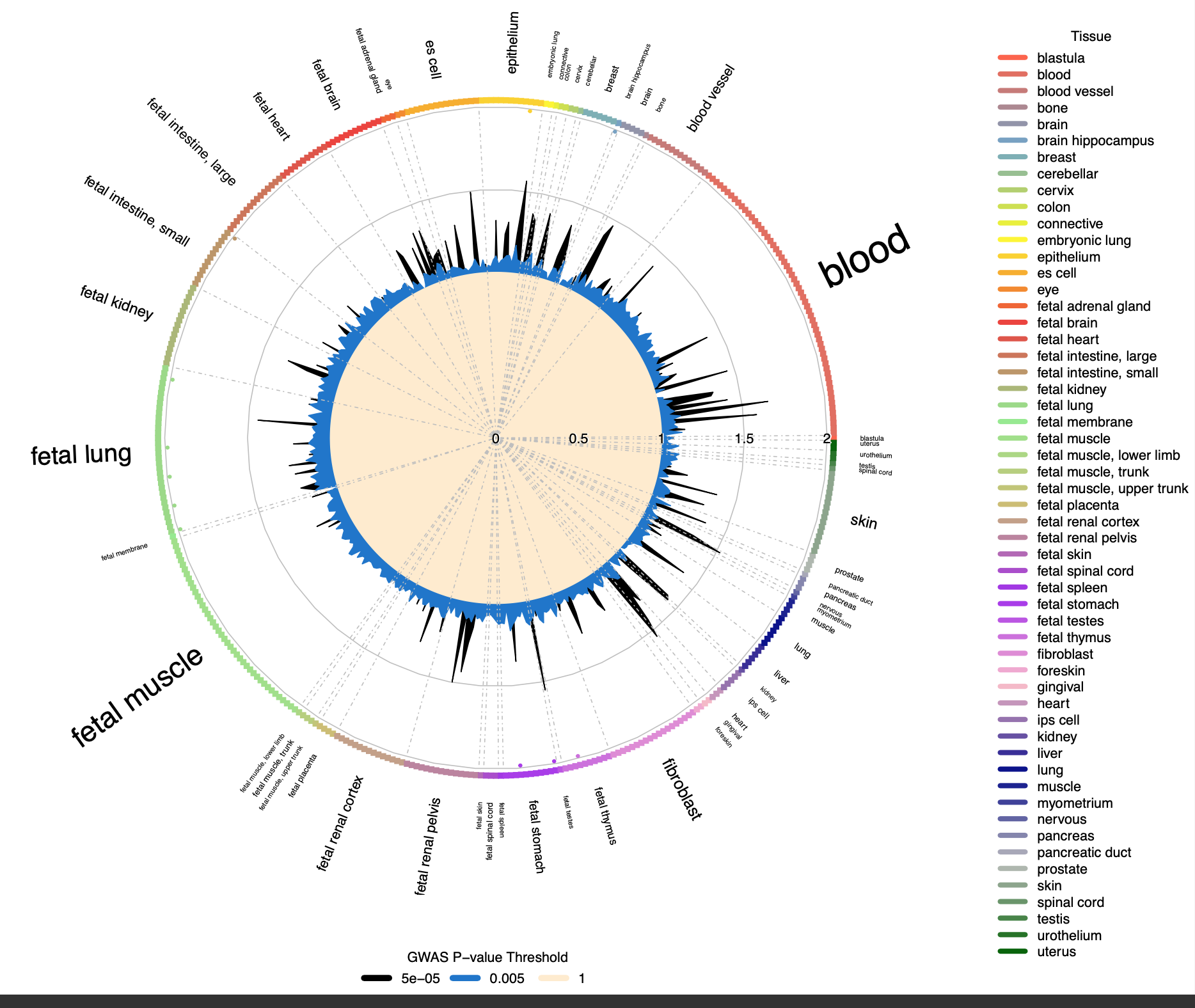
**

## Figure S8. Effect of SES adjustment on genome-wide signals


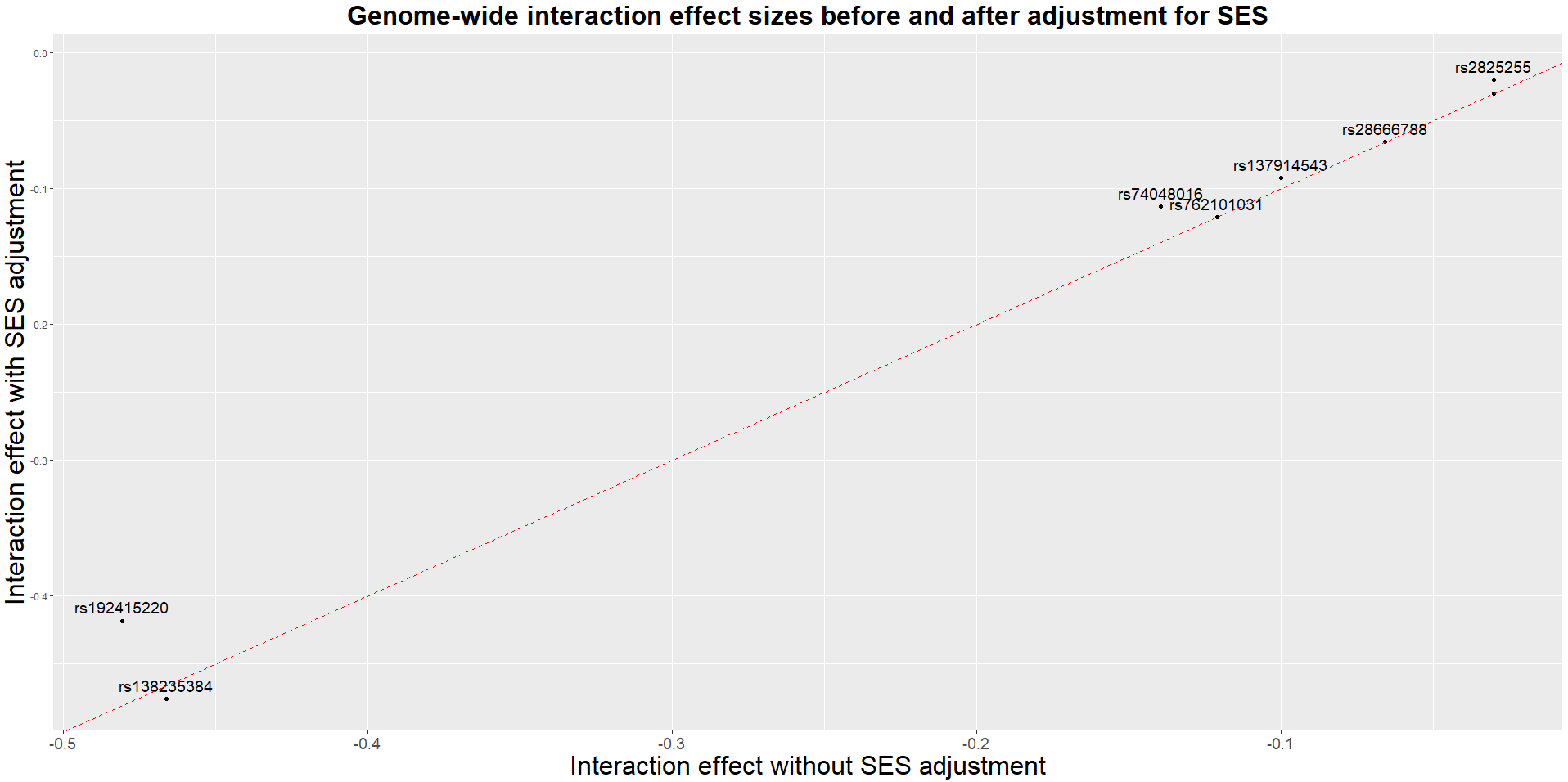


Figure S9. Interaction effects stratified by education group for (A) rs137914543, rs138235384, rs192415220, rs28666788, rs74048016, rs762101031 (B) rs2825255 (FEV_1_ and FVC phenotypes)

SD = standard deviation, Education group is coded as 0 - Lower vocational qualification or less and 1 – Higher vocational qualification or more, Interaction effect is per 10 μg/m^3^ increase in air pollutant NO_2_  and per 5 μg/m^3^ increase for air pollution variables PM_10_ and PM_2.5_ as the coded allele increases.

##


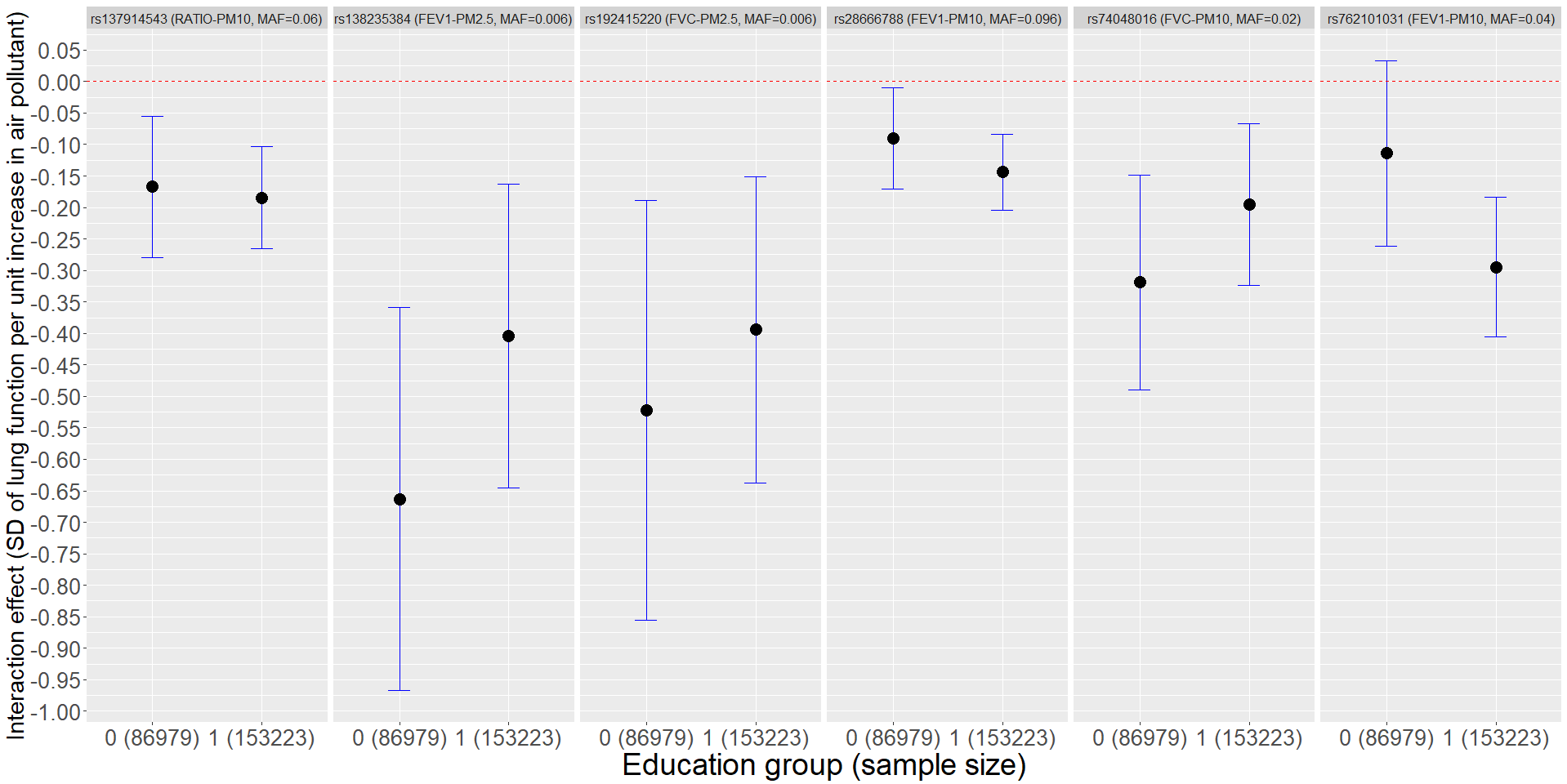
(A)

(B)


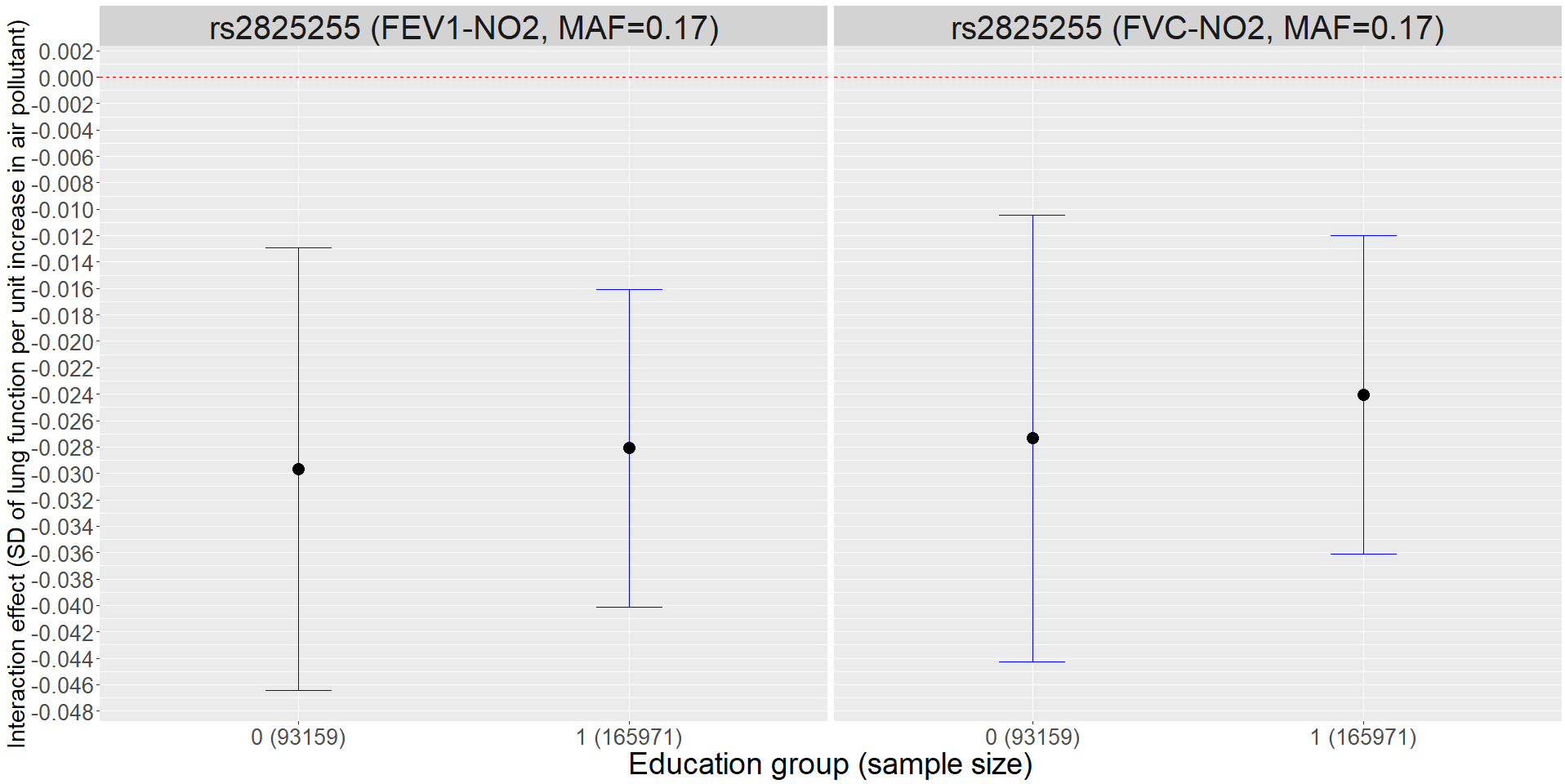


Figure S10. Interaction effects stratified by income group for (A) rs74048016 (B) rs28666788 (C) rs137914543 (D) rs762101031 (E) rs192415220 (F) rs138235384 (G) rs2825255 (for FEV_1_ phenotype) (H) rs2825255 (for FVC phenotype)

SD – Standard Deviation, Income group is coded as 0 - lowest income group to 5 - highest income group, Interaction effect is per 10 μg/m^3^ increase in air pollutant NO_2_  and per 5 μg/m^3^ increase for air pollution variables PM_10_ and PM_2.5_ as the coded allele increases.

1.
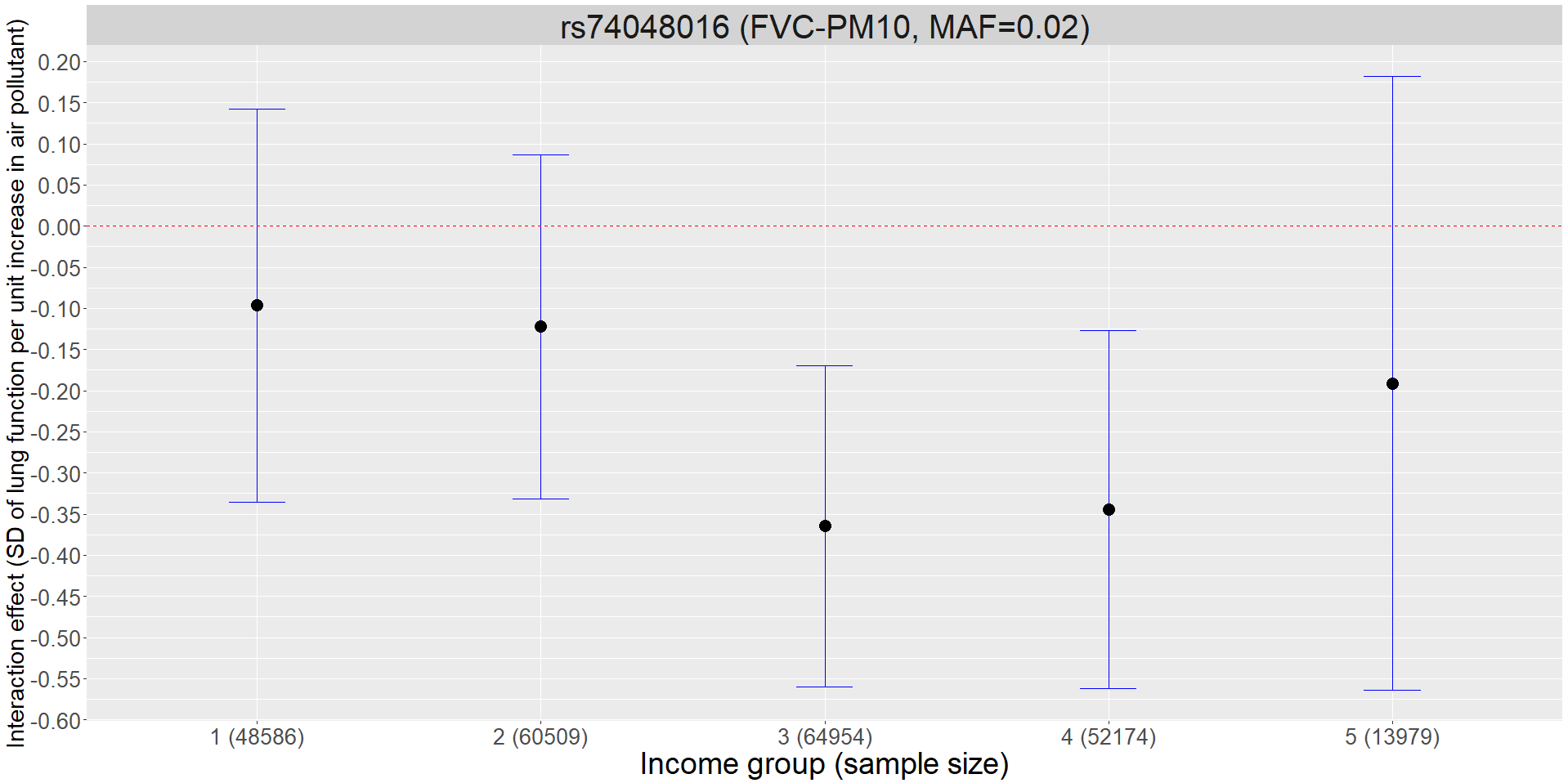


##


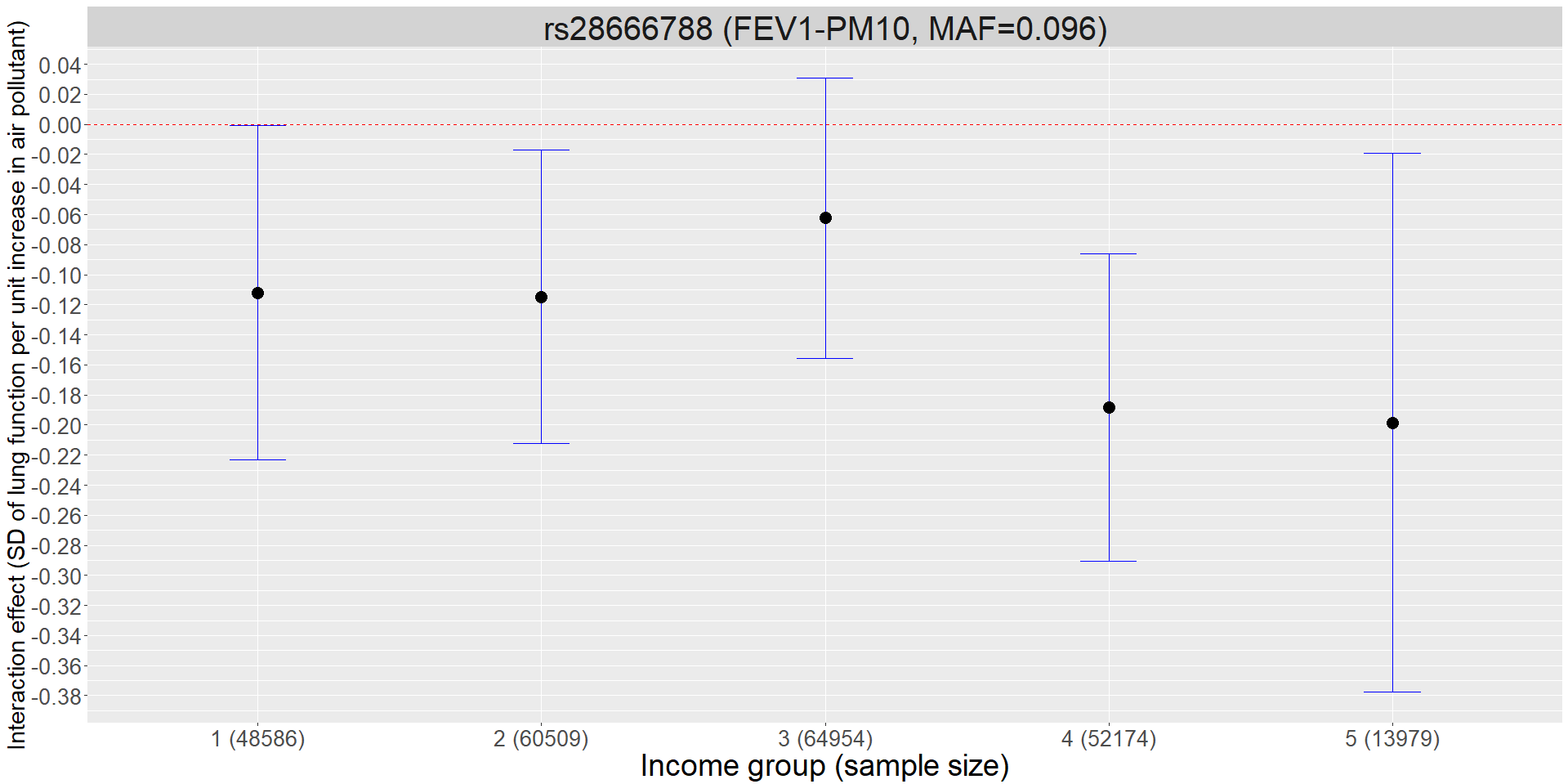


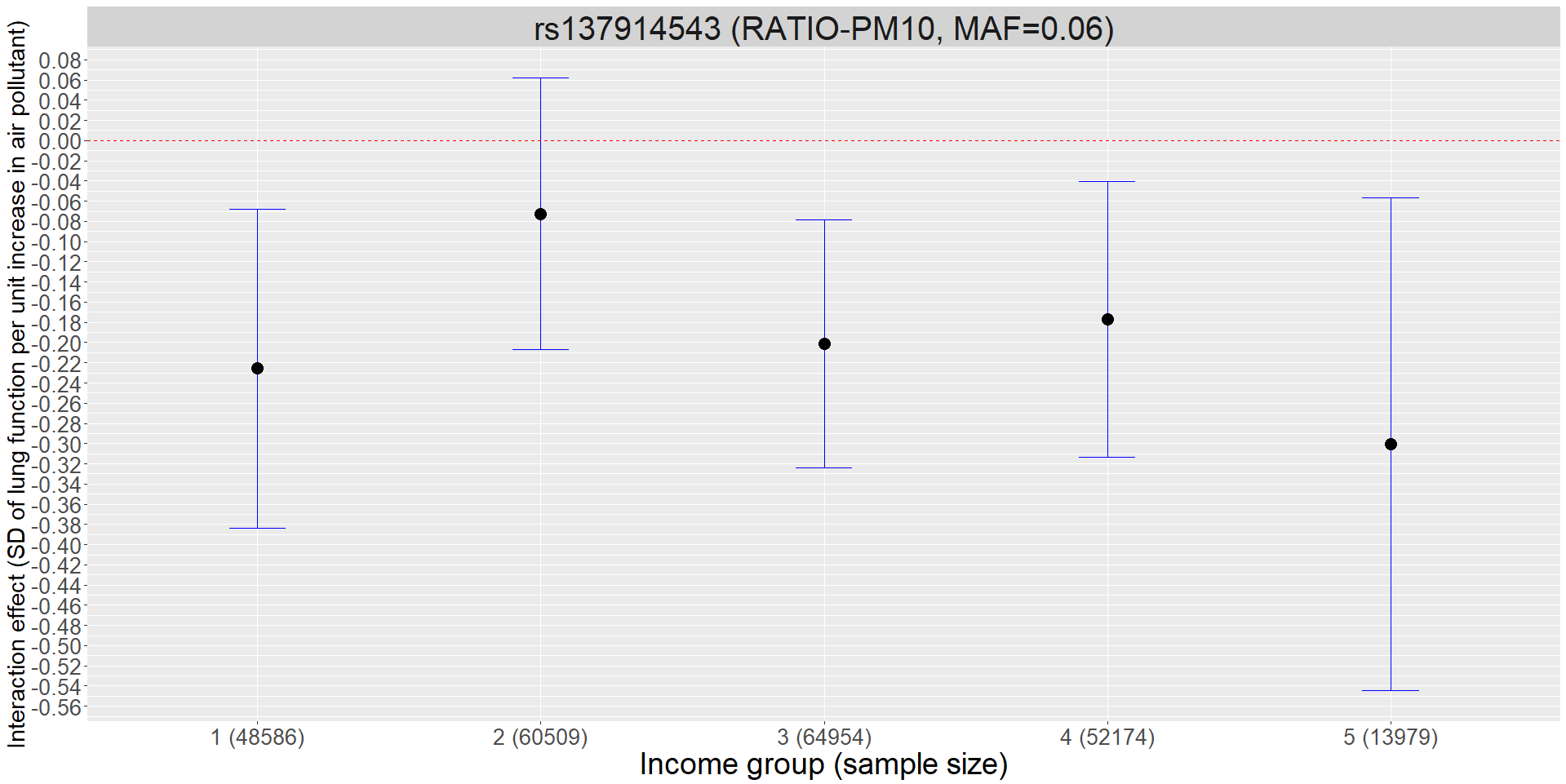


1.
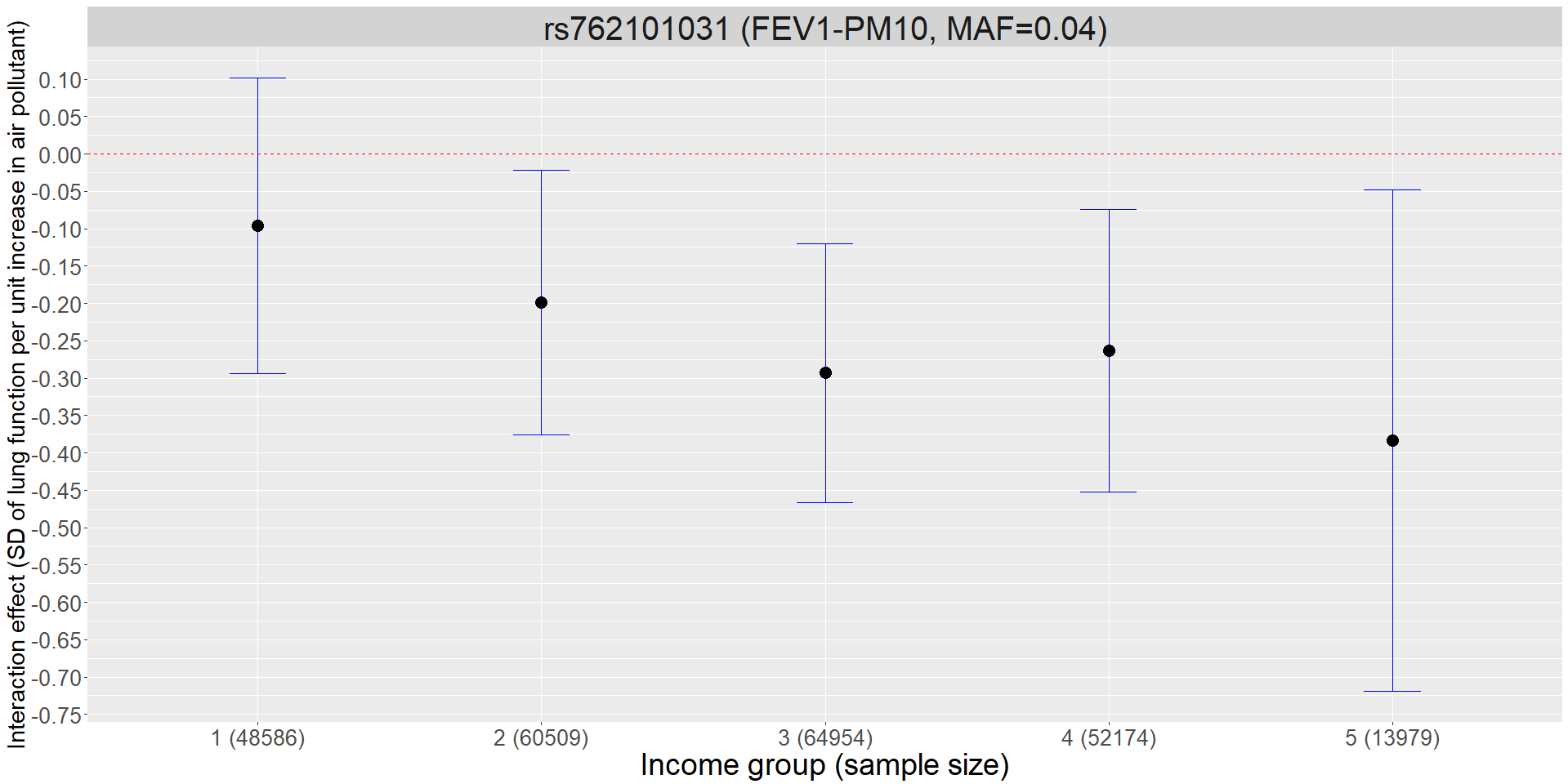


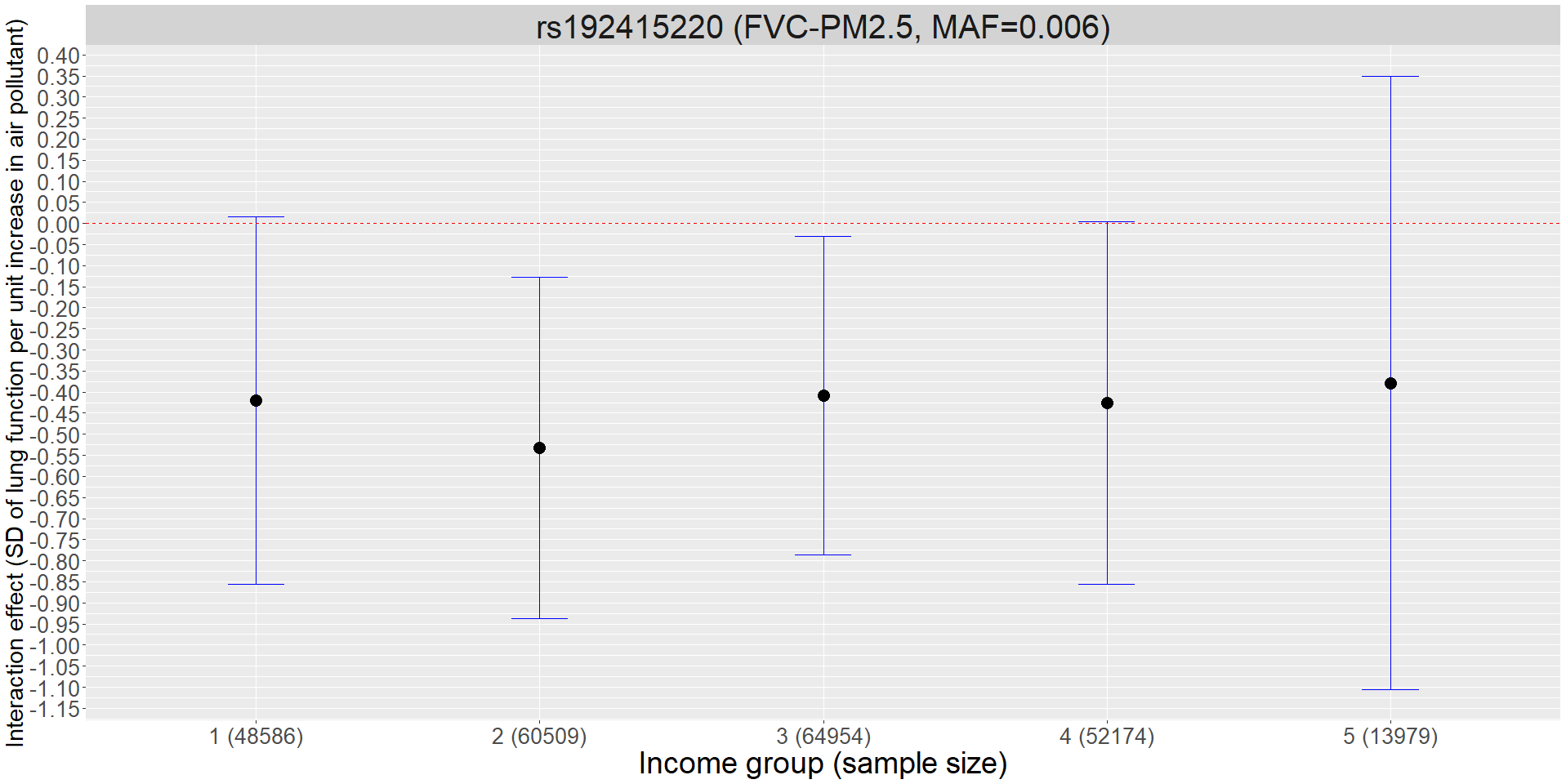


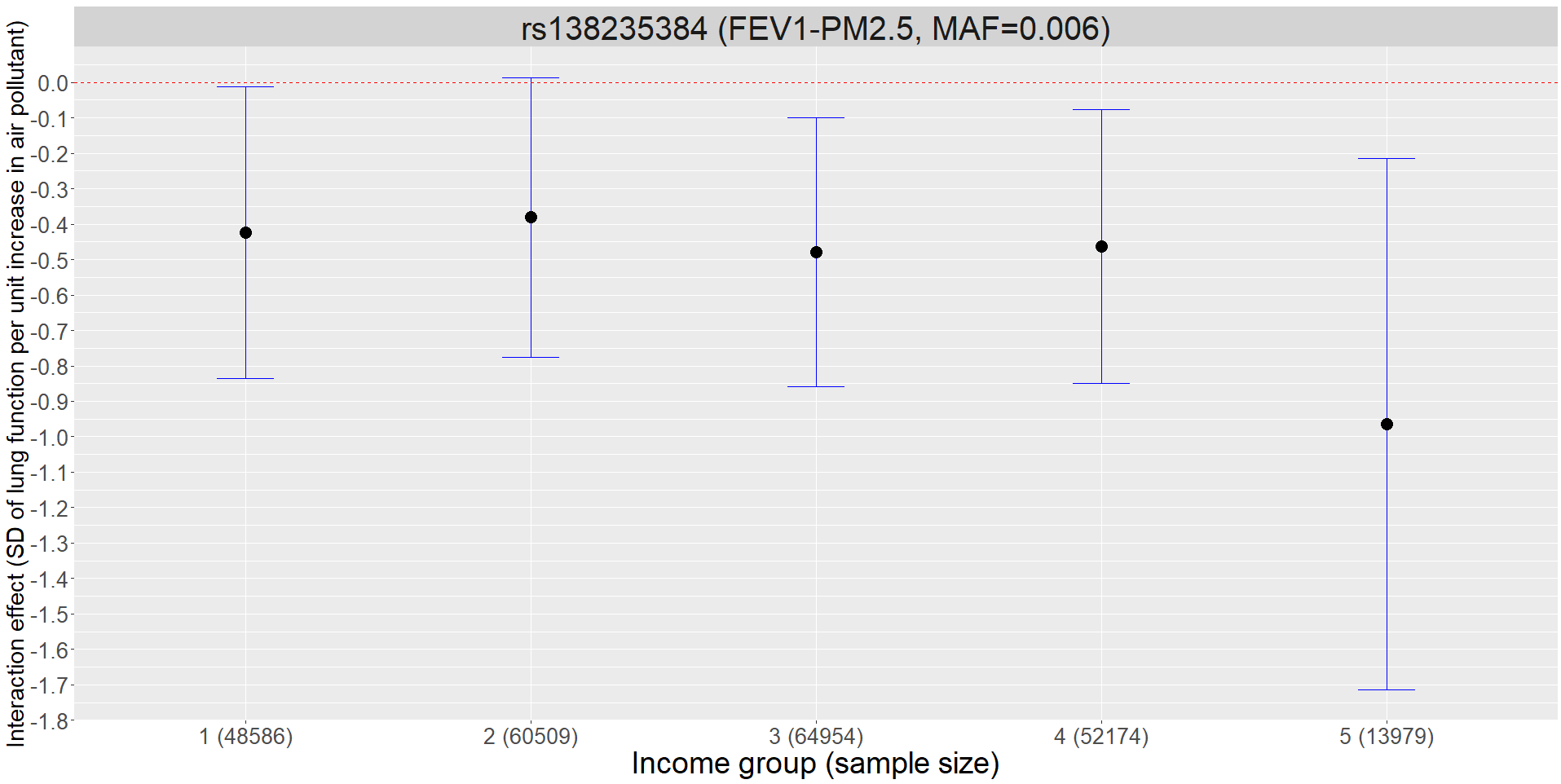


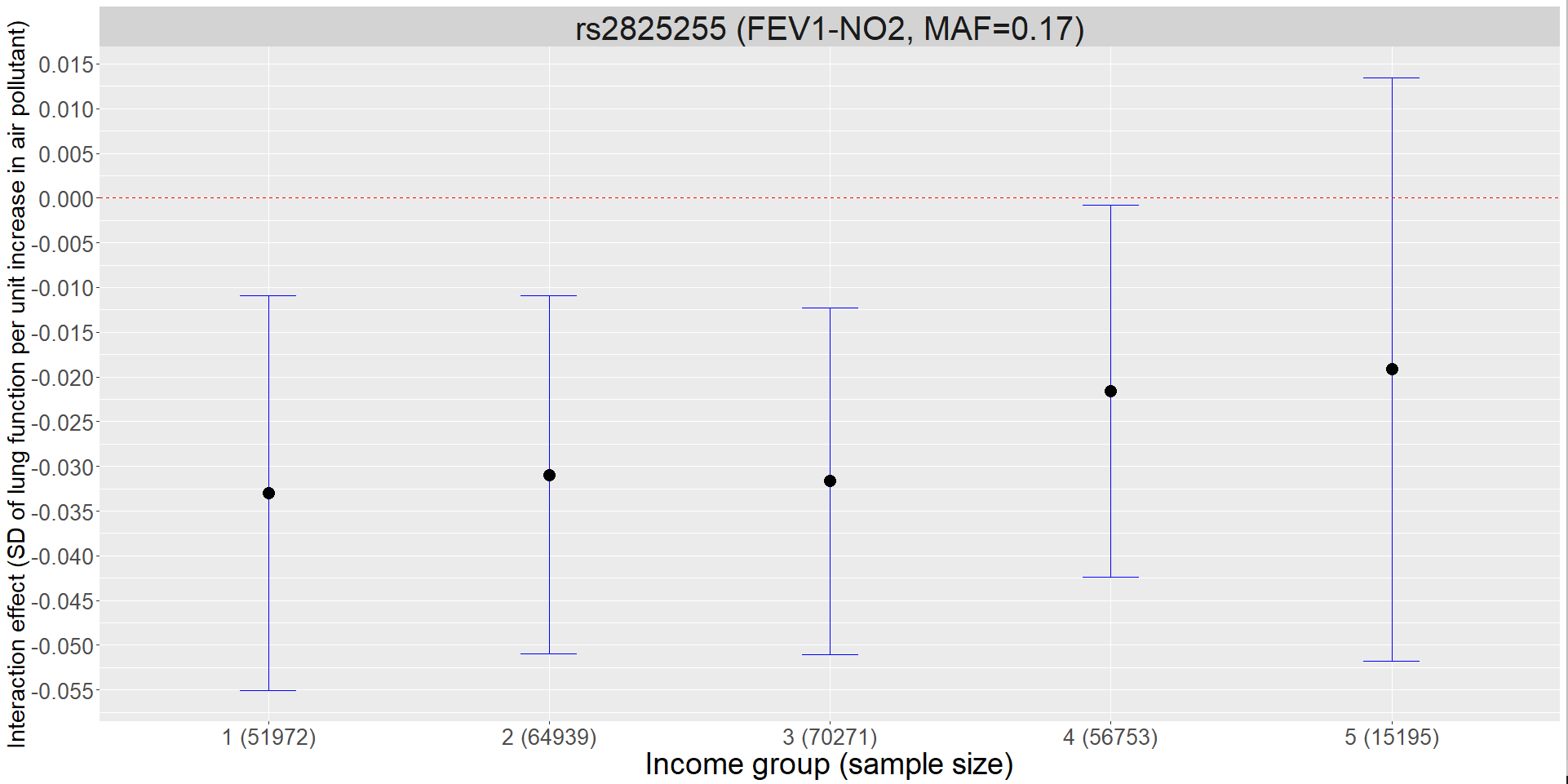


1.
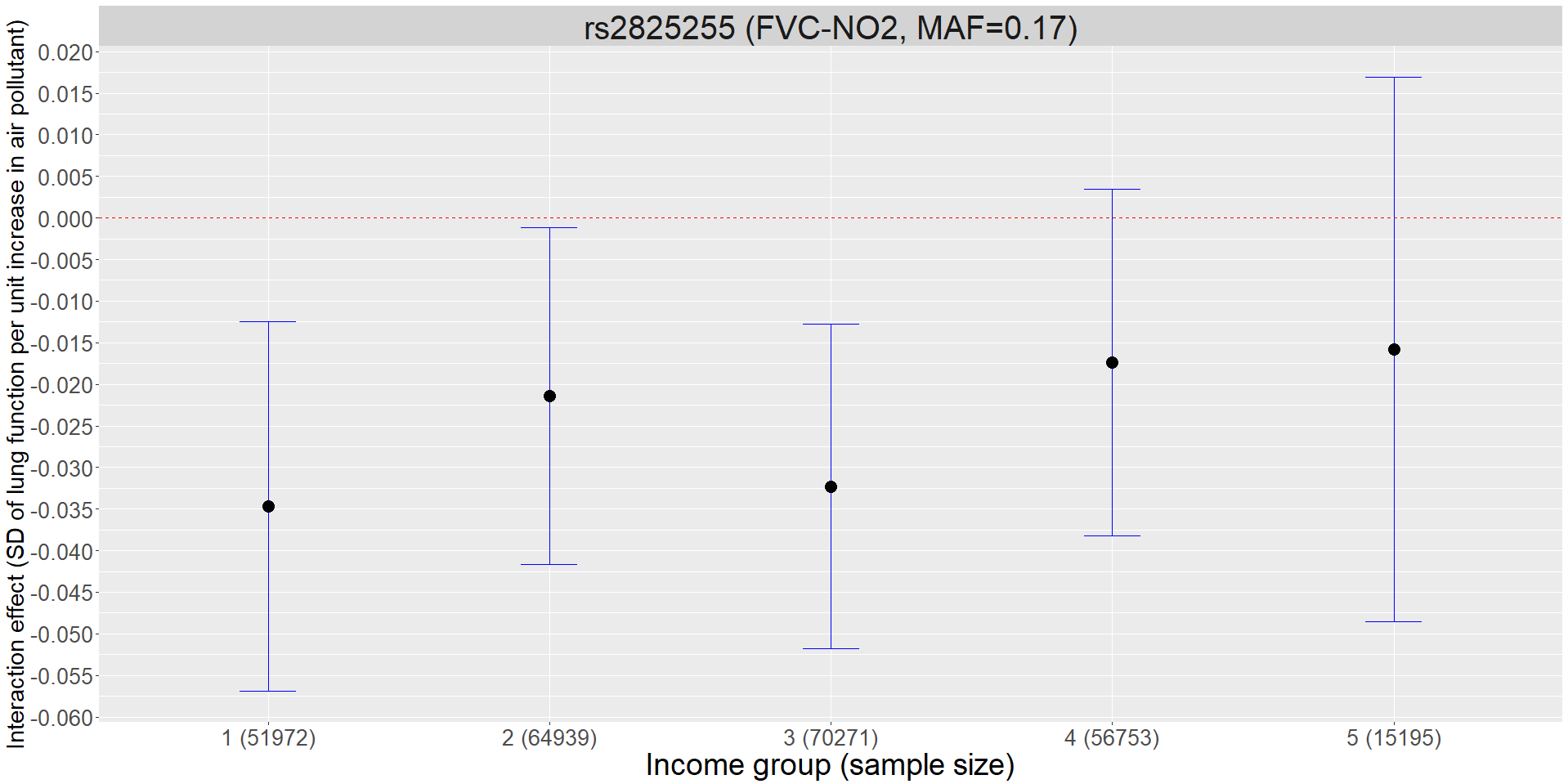

Supplement: Supplementary data 2 [file mmc2.docx]
